# Discovery of Inhibitors of *Trypanosoma brucei* by Phenotypic Screening of a Focused Protein Kinase Library

**DOI:** 10.1002/cmdc.201500300

**Published:** 2015-09-18

**Authors:** Andrew Woodland, Stephen Thompson, Laura A T Cleghorn, Neil Norcross, Manu De Rycker, Raffaella Grimaldi, Irene Hallyburton, Bhavya Rao, Suzanne Norval, Laste Stojanovski, Reto Brun, Marcel Kaiser, Julie A Frearson, David W Gray, Paul G Wyatt, Kevin D Read, Ian H Gilbert

**Affiliations:** [a] Drug Discovery Unit, Division of Biological Chemistry and Drug Discovery, University of DundeeDundee, DD1 5EH (UK) E-mail: i.h.gilbert@dundee.ac.uk; [b] Swiss Tropical and Public Health InstituteSocinstrasse 57, P.O. Box, 4002, Basel (Switzerland)

**Keywords:** cidal, kinases, phenotypic, static, *Trypanosoma brucei*

## Abstract

A screen of a focused kinase inhibitor library against *Trypanosoma brucei rhodesiense* led to the identification of seven series, totaling 121 compounds, which showed >50 % inhibition at 5 μm. Screening of these hits in a *T. b. brucei* proliferation assay highlighted three compounds with a 1*H*-imidazo[4,5-*b*]pyrazin-2(3*H*)-one scaffold that showed sub-micromolar activity and excellent selectivity against the MRC5 cell line. Subsequent rounds of optimisation led to the identification of compounds that exhibited good in vitro drug metabolism and pharmacokinetics (DMPK) properties, although in general this series suffered from poor solubility. A scaffold-hopping exercise led to the identification of a 1*H*-pyrazolo[3,4-*b*]pyridine scaffold, which retained potency. A number of examples were assessed in a *T. b. brucei* growth assay, which could differentiate static and cidal action. Compounds from the 1*H*-imidazo[4,5-*b*]pyrazin-2(3*H*)-one series were found to be either static or growth-slowing and not cidal. Compounds with the 1*H*-pyrazolo[3,4-*b*]pyridine scaffold were found to be cidal and showed an unusual biphasic nature in this assay, suggesting they act by at least two mechanisms.

## Introduction

Human African trypanosomiasis (HAT), or sleeping sickness, as it is more commonly known, is an often fatal infection endemic in sub-Saharan Africa. Current treatments for HAT are inadequate due to toxicity and inappropriate means of administration for remote rural settings, where the disease is predominantly found. Fortunately, the number of cases of HAT appears to be decreasing; however, there is an urgent need for new effective treatments for HAT, to improve treatment and to facilitate the eventual eradication of this disease. HAT is caused by the protozoan parasites *Trypanosoma brucei gambiense* and *T. b. rhodesiense*. The parasites have a complex life cycle, involving the human host and the tsetse fly, which is the vector. Initially there is a peripheral infection in which the parasite is predominantly found in the blood. However, the parasites eventually invade the central nervous system, which can lead to coma and death.[1,2]

Protein kinases are a major drug target for human diseases, particularly oncology.[3] Analysis of the *T. brucei* genome indicates that there are 156 protein kinases in the trypanosome kinome.[4–6] Whilst parasite protein kinases have been relatively poorly investigated, there appear to be significant differences from human kinases. In particular:

- A relatively large number of protein kinases associated with cell-cycle control, which may be a consequence of the complex life cycle of the parasite;
- A lack of tyrosine kinases (except dual-specificity kinases);
- A number of unique kinases.

In addition, a number of protein kinases have been genetically validated as potential drug targets in *T. brucei* using RNA interference (RNAi) or gene knockouts.[4,5,7–9] Given that protein kinases are generally known to be druggable, they represent promising drug targets in trypanosomatids. The Drug Discovery Unit (DDU) in Dundee has recently carried out screens and chemical optimisation programmes against several parasite protein kinases. Although we have prepared compounds with significant (low-nanomolar) activity against the proteins, this did not always translate into potent cellular activity.[10]

A complementary method to target-based drug discovery is phenotypic drug discovery,[11] which has several advantages. Firstly, all possible drug discovery targets are present in their natural environment, allowing an unbiased and more physiologically relevant screening platform; this may give rise to compounds that inhibit more than one target. Indeed it has been found in the oncology field that compounds that inhibit more than one protein kinase are often required for activity. Secondly, as the primary screening platform is a functional efficacy screen, the relationship between target and phenotype does not need to be established. Finally, compounds must be able to penetrate cells and have a sufficient free fraction in the assay to elicit their response, eliminating compounds with inappropriate properties.[12–14] We therefore decided to conduct a phenotypic screen of a focused kinase compound library against whole parasites. A similar exercise was recently reported by Diaz et al., in which a phenotypic screen of a kinase-targeted library from GlaxoSmithKline (GSK) was reported and gave rise to a number of actives.[15] There is also a recent report of a large screen against kinetoplastids with 1.8 million compounds from GSK.[16] The ideal target product profile to treat HAT requires a compound that can treat both stage 1 (peripheral) and stage 2 (CNS) infection;[8] thus the compound should have blood–brain barrier (BBB) permeability.

## Results and Discussion

### The focused screen

The Dundee focused protein kinase library,[17] which at that point contained 3885 compounds, was assayed by the Swiss Tropical and Public Health Institute (STPH) against *T. b. rhodesiense* at 1 and 5 μm. From this original triage, seven series, totaling 121 compounds, were identified which showed >50 % inhibition of parasite growth at 5 μm. These were progressed into EC_50_ determination in a *T. b. brucei* proliferation assay and assessed in a MRC5 proliferation assay to provide an early indicator of toxicity to mammalian cells. From this, seven compounds showed EC_50_ values <1 μm against *T. b. brucei*. The most active hit compound was a 1*H*-imidazo[4,5-*b*]pyrazin-2(3*H*)-one (**1**), which had an EC_50_ value of 80 nm against *T. b. brucei* and was nontoxic to the mammalian MRC5 cell line (EC_50_>50 μm). Two additional 1*H*-imidazopyrazinone analogues had EC_50_ values of <1 μm against *T. b. brucei* (Table 1). The physicochemical properties were calculated in StarDrop (http://www.optibrium.com). It has been proposed that for a compound to have BBB permeability, it should have a topological polar surface area (tPSA) of <90 Å^2^ and a molecular weight (*M*_r_) less than 450 Da.[18] Wager's research group have proposed more sophisticated models.[19–21] The *M*_r_ and tPSA values were all within the preferred range for a potential CNS-penetrant compound. The calculated log*P* values were also in the range of CNS-penetrant compounds.[19] Based on the initial data we decided to progress the project into hits-to-leads development.

**Table 1:** Potency of hits 1–3

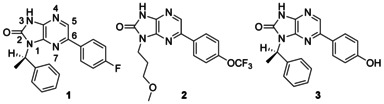					
ID	clog*P*	tPSA [Å^2^]	*M*_r_ [Da]	EC_50_ [μm]^[a]^	
				*T. b. brucei*	MRC5
**1**	3.3	57	334	0.08	>50
**2**	2.8	75	370	0.5	15
**3**	2.9	77	334	0.9	ND

[a] Values are the geometric mean of two or more determinations; standard deviation is typically within 2-fold of the EC_50_ value. ND: not determined.

### Hit validation

**Scheme 1:**
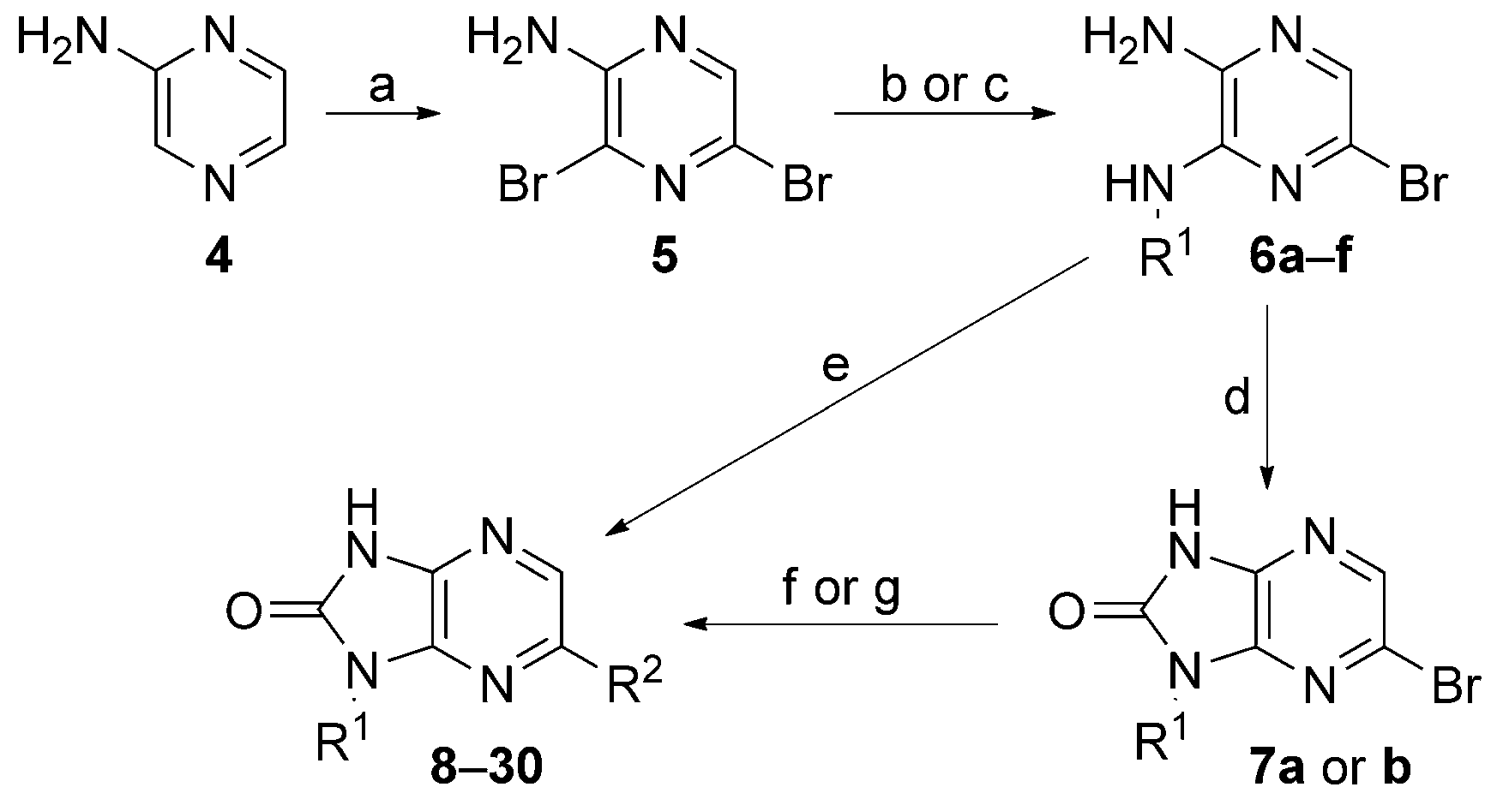
*Reagents and conditions*: a) NBS, MeCN, 18 h, RT, 12 %; b) amine, DIPEA, EtOH, 180 °C (microwave), 30 min, 27–64 %; c) cyclohexylamine, DIPEA, *n*BuOH, 220 °C (microwave), 30 min, 95 %; d) CDI, 1,4-dioxane, 80 °C, 16 h, 31 %; e) for 8–11, 13–15, 17, and 18: 1) Pd(PPh_3_)_4_ (3 mol %), K_2_CO_3_ (1.5 m aqueous), boronic acid, DMF, 140 °C, 5 min; 2) CDI, 1,4-dioxane, 140 °C, 20 min, 3–34 %; f) for 12 and 19–30: Pd(PPh_3_)_4_ (3 mol %), K_2_CO_3_ (1.5 m aqueous), boronic acid, DMF, 140 °C, 5 min, 7–67 %; g) for 16: 7, H_2_, 5 % Pd/C, MeOH, 15 %.

Medicinal chemistry was initiated with the aim of validating and profiling the hit series and increasing the potency of the compounds. The desired 1*H*-imidazopyrazinones were synthesised in four steps from 2-aminopyrazine **4**. Bromination with NBS gave the versatile scaffold 2,5-dibromo-4-aminopyrazine **5**. Regioselective displacement of the 5-bromine group with amines could be conducted using microwave heating to give intermediates **6 a**–**f** (Scheme 1). Compounds **8**–**11**, **13**–**15**, **17**, and **18** were prepared by Suzuki reaction with the appropriate diamine precursor followed by cyclisation with CDI. Cyclisation of the appropriate diamine (**6 a** or **b**) with CDI gave **7 a** or **7 b**, which were then reacted under Suzuki conditions to give compounds **12** and **19**–**30**. Treatment of **7 a** under hydrogenation conditions afforded compound **16**.

Enantiomers **1** and **8** were equipotent, whilst removal of the benzylic methyl group (compound **9**) resulted in a fivefold decrease in activity (Table 2). Replacement of the benzyl group with methylcyclopropane (compound **10**) had little effect on the activity, whilst further truncation of the amine to a methyl group (in **11**) resulted in a 100-fold drop in activity relative to methyl cyclopropane (in **10**). Replacement of the methylbenzyl group of **1** with a methoxypropyl group (in **13**) resulted in a sevenfold loss in activity. Interestingly, replacement of the methylbenzyl group of **1** with cyclohexane (compound **12**) resulted in a threefold improvement in activity. Compounds showed good selectivity for the parasite over MRC5 cells.

**Table 2:** Variation of the R^1^ group of compound 1

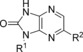						
ID	R^1^	R^2^	clog*P*	EC_50_ [μm]^[a]^		Aq. Sol.
				*T. b. brucei*	MRC5	[μm]^[b]^
**1**	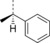	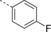	3.6	0.08	>50	<12
**8**	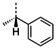	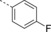	3.6	0.09	>50	ND
**9**	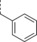	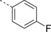	3.3	0.5	>50	ND
**10**	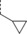	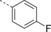	2.7	0.3	>50	ND
**11**	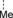	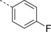	1.9	40	>50	ND
**12**	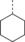	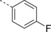	3.9	0.03	>50	55
**13**	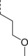	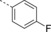	2.0	0.59	>50	ND

[a] Values are the geometric mean of two or more determinations; standard deviation is typically within 2-fold of the EC_50_ value. [b] Aqueous kinetic solubility. ND: not determined.

The 4-fluorophenyl group of **1** could be replaced with a phenyl ring (in **14**) with no loss of activity (Table 3). The larger 4-trifluoromethyloxy analogue **15** was 10-fold less active than **1**, but was comparable with the hit compound **2**, further demonstrating that changes to the R^1^ group were tolerated. Replacement of the phenyl ring of **14** with a hydrogen atom in **16** resulted in a >300-fold loss of activity relative to **14**. Substitution of the phenyl ring with a 3-fluoro group was tolerated in **17**, while the larger and more polar 3-methanesulfonamide group (in **18**) resulted in a 30-fold loss of activity.

**Table 3:** Variation of the R^2^ group of compound 1

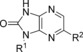						
ID	R^1^	R^2^	clog*P*	EC_50_ [μm]^[a]^		Aq. Sol.
				*T. b. brucei*	MRC5	[μm]^[b]^
**1**	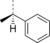	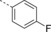	3.6	0.08	>50	<12
**14**	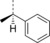	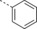	3.5	0.1	26	<12
**15**	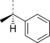	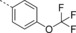	4.0	1	39	ND
**16**	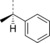	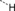	2.0	36	>50	ND
**17**	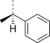	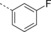	3.6	0.3	44	ND
**18**	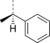	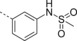	2.5	3	46	ND

[a] Values are the geometric mean of two or more determinations; standard deviation is typically within 2-fold of the EC_50_ value. [b] Aqueous kinetic solubility. ND: not determined.

### Kinase panel and DMPK data

These data validated the series, as there was a definite structure activity relationship, and several examples demonstrated sub-100 nm EC_50_ values in the *T. b. brucei* proliferation assay. The compounds also showed excellent selectivity over human MRC5 cells. Consequently, it was decided to profile the compounds further for potential inhibition of human kinases and to study their DMPK properties to ensure that there were no major issues which may impact further development.

The DDU kinase-focused compound set contains lead-like scaffolds that are designed to target protein kinases; they have kinase hinge binding motifs. Four of the 1*H*-imidazopyrazinones were screened against a human/mammalian kinase panel at the University of Dundee (http://www.kinase-screen.mrc.ac.uk) at a concentration of 10 μm. At the time of testing the panel contained 79 mammalian kinases selected to provide a broad coverage of the mammalian kinome (∼500 kinases in the human genome).[3] None of the compounds were active against any of the kinases at 10 μm. Although the results did not provide any clues as to the molecular target in the parasite, they did show the 1*H*-imidazopyrazinones to have promising selectivity over mammalian kinases.

Two key compounds, **1** and **12**, showed low in vitro mouse hepatic microsomal intrinsic clearance (*CL*_int_ <0.5 and 0.7 mL min^−1^ g^−1^, respectively), consistent with good metabolic stability. Compound **1**, with promising in vitro anti-parasitic activity, low toxicity to human cells, and promising in vitro metabolic stability, was progressed into in vivo pharmacokinetic (PK) determination. After a single oral dose at 5 mg kg^−1^ to female NMRI mice, the compound showed a low *C*_max_ value and took approximately 2 h to reach *C*_max_, both of which are suggestive of solubility-limited absorption (Table 4). Furthermore, **1** showed high protein binding (0.3 % unbound). Consequently, the observed free blood concentration was likely to be too low for the compound to be efficacious in vivo even at high dose, so further development was required to improve free oral exposure for **1**. Common strategies to address the key issues of poor solubility and/or high protein binding include decreasing the lipophilicity (log*P*), addition of solubilising groups, and reducing the planarity of the molecule to minimise crystal stacking.[22–25]

**Table 4:** PK data for compound 1

Route	Dose [mg kg^−1^]	*C*_max_ [ng mL^−1^]^[a]^	*t*_max_ [h]^[b]^	AUC_(0–8)_ [ng min mL^−1^]^[c]^	*t*_1/2_ [h]^[d]^
p.o.	5	110	4	36 000	3

[a] Maximum concentration reached. [b] Time after initial dose at which *C*_max_ was reached. [c] Area under the curve. [d] Half-life of compound in the blood.

### Hit to leads

#### The R^2^ position

A round of optimisation for the R^2^ position was commenced. During this process R^1^ was retained as a cyclohexyl moiety, as this substituent gave the greatest activity and, due to its non-planar structure, should also increase the solubility of the compounds. A variety of substituents were investigated at the *para*, *meta*, and *ortho* positions of the phenyl group at the R^2^ position. The substituents were designed either to disrupt the packing and/or to reduce lipophilicity. Compounds were prepared using the route described in Scheme 1 (Table 5).

**Table 5:** Variation of the R^3^, R^4^, and R^5^ groups of compound 19

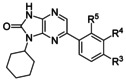								
ID	R^3^	R^4^	R^5^	PPB	clog*P*	EC_50_ [μm]^[a]^		Aq. Sol.
				[% free]		*T. b. brucei*	MRC5	[μm]^[b]^
**19**	H	H	H	0.8	3.9	0.04	>50	110
**20**	OMe	H	H	0.7	3.8	0.33	24	ND
**21**	CH_2_OH	H	H	4.7	2.9	0.4	>50	ND
**22**	C(O)NHMe	H	H	ND	2.9	1.7	>50	ND
**23**	CH_2_NMe_2_	H	H	ND	3.8	5	>50	ND
**24**	H	CH_2_OH	H	ND	2.9	0.03	>50	>250
**25**	H	NHCOMe	H	ND	3.2	0.9	>50	ND
**26**	H	H	F	0.7	3.9	0.06	>50	20
**27**	H	H	OMe	ND	3.9	0.9	>50	ND
**28**	H	H	CH_2_OH	7.2	2.9	0.05	>50	>250

[a] Values are the geometric mean of two or more determinations; standard deviation is typically within 2-fold of the EC_50_ value. [b] Aqueous kinetic solubility. ND: not determined.

For the *para*-substituted compounds, the electron-donating methoxy group in **20** showed an eightfold drop in activity. The introduction of polar groups was poorly tolerated, with hydroxymethyl compound **21** being 10-fold less active, while amide **22** and dimethylaminomethyl compound **23** were 30- and 50-fold less active, respectively. Substituents at the *para* position generally caused a drop in activity, relative to those in the *ortho* or *meta* positions. Encouragingly, the hydroxymethyl substituents retained activity in the *ortho* and *meta* positions (**28** and **24**, respectively) and gave rise to good solubility.

**Scheme 2:**
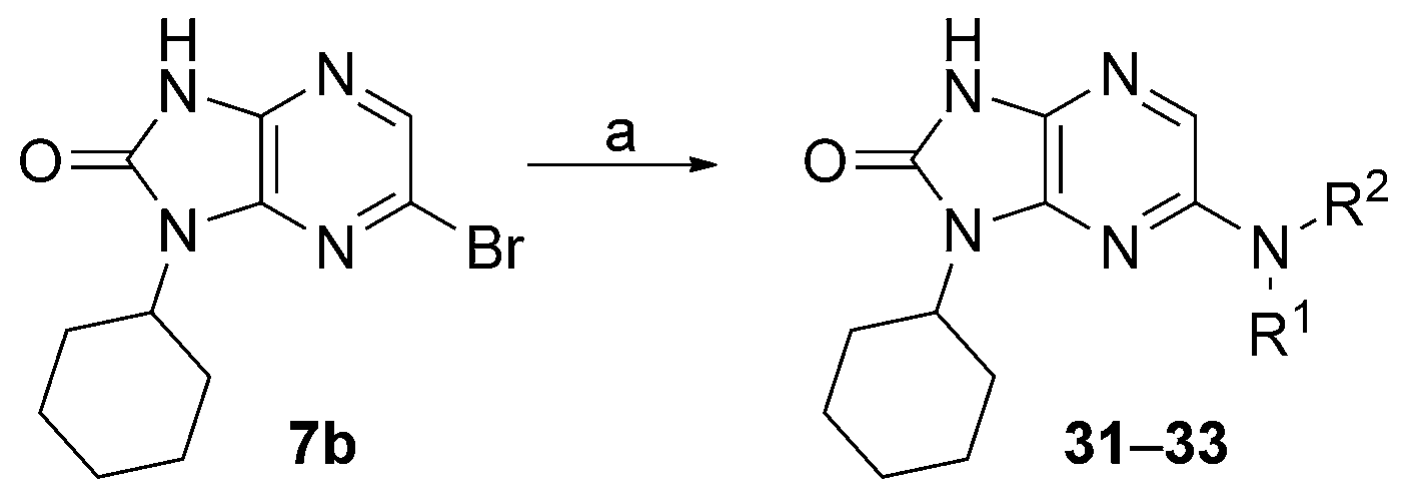
*Reagents and conditions*: a) Pd_2_dba_3_ (5 mol %), amine (2 equiv), *rac*-binap (10 mol %), *t*BuOK (3 equiv), 1,4-dioxane, 160 °C (microwave), 20 min, 5–24 %.

Further work was undertaken in which the phenyl ring of **19** was replaced with a heterocycle or saturated ring system, which should increase solubility. For the introduction of amines we used a Buchwald reaction on intermediate **7 b** (Scheme 2). For the introduction of aromatic heterocycles we used the chemistry described above. Both 4-pyridyl **29** and 3-pyridyl **30** compounds were equipotent to phenyl **19**, demonstrating a tolerance for a hydrogen bond acceptor at the 3- and 4-positions (Table 6). Both piperidine **31** and the more polar morpholine **32** were tolerated, but the basic piperazine **33** was 50-fold less active than the phenyl compound **19**.

**Table 6:** Variation of the R^2^ group of compound 19

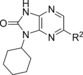					
ID	R^2^	clog*P*	EC_50_ [μm]^[a]^		Aq. Sol.
			*T. b. brucei*	MRC5	[μm]^[b]^
**19**	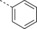	3.9	0.04	>50	110
**29**	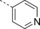	3.0	0.07	>50	ND
**30**	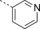	3.0	0.1	>50	ND
**31**	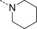	3.5	0.08	>50	ND
**32**	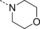	2.3	0.05	>50	220
**33**	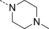	2.3	2	>50	ND

[a] Values are the geometric mean of two or more determinations; standard deviation is typically within 2-fold of the EC_50_ value. [b] Aqueous kinetic solubility. ND: not determined.

#### The R^1^ position

**Scheme 3:**
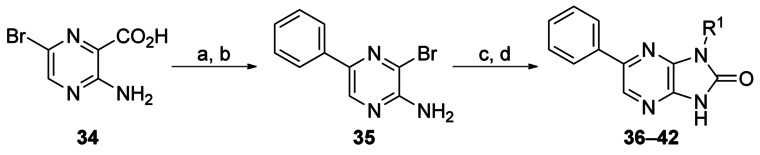
*Reagents and conditions*: a) phenylboronic acid, Pd(PPh_3_)_4_, K_2_CO_3_ (2 m aqueous), DMF, 140 °C (microwave), 10 min, 100 %; b) NaOAc, AcOH, Br_2_, RT, 16 h, 63 %; c) amine, DIPEA, *n*BuOH, 220 °C (microwave), 1 h; d) CDI, dioxane, 130 °C (microwave), 10 min, 2–71 % over two steps.

The initial rounds of medicinal chemistry demonstrated that changes to the R^1^ group were tolerated and that the cyclohexyl ring of **12** was slightly more active than the initial α-methylbenzyl group of **1**. To complete the exploration of the R^1^ group, an optimisation array was conducted, maintaining a phenyl ring at the R^2^ position for reference. 3-Bromo-5-phenylpyrazine-2-amine **35** was prepared from 3-amino-6-bromopyrazine-2-carboxylic acid **34** in two steps (Scheme 3). Displacement of the 3-bromo group followed by cyclisation with CDI gave compounds **36**–**42**. Truncation of **1** to an isopropyl group (in **36**) resulted in a 10-fold drop in activity while rigidification by replacement of cyclohexyl with an aromatic ring (in **37**) resulted in a complete loss of activity (Table 7). The majority of changes were designed to introduce polar solubilising groups. Alkyl ethers **38** and **39** showed only two- and fivefold losses of activity, respectively. Alkoxy compound **40** was ∼10-fold less active than the cyclohexyl, whereas the basic compounds **41** and **42** showed poor antitrypanosomal activity.

**Table 7:** Variation of the R^1^ group of compound 19

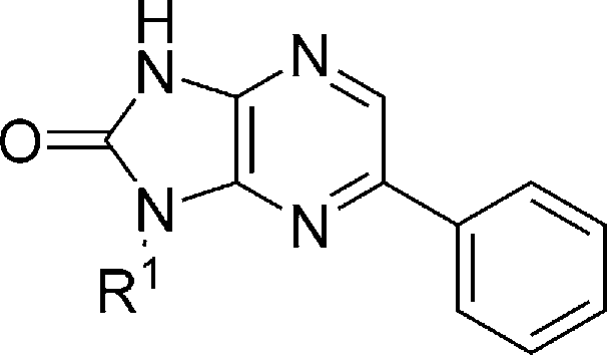					
ID	R^1^	clog*P*	EC_50_ [μm]^[a]^		Aq. Sol.
			*T. b. brucei*	MRC5	[μm]^[b]^
**19**	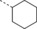	3.9	0.04	>50	110
**36**	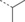	2.5	0.3	>50	ND
**37**	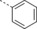	2.9	>50	>50	ND
**38**	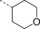	2.3	0.1	>50	55
**39**	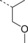	2.0	0.2	>50	ND
**40**	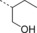	1.7	0.5	>50	ND
**41**	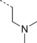	1.7	13	>50	ND
**42**	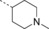	2.4	2	>50	ND

[a] Values are the geometric mean of two or more determinations; standard deviation is typically within 2-fold of the EC_50_ value. [b] Aqueous kinetic solubility. ND: not determined.

Both **28** and **38** showed decreased protein binding relative to **1** (7.2 and 15 % unbound, respectively, versus 0.3 % unbound) and improved solubility (>250 and 55 μm, respectively, compared with <12 μm). However, both compounds were less metabolically stabile when incubated with mouse hepatic microsomes (*CL*_int_=2.4 and 1.6 mL min^−1^ g^−1^, respectively, versus <0.5 mL min^−1^ g^−1^) but still progressable. Given these results, both **28** and **38** were progressed into in vivo PK studies. Compound **28** showed significantly higher levels of exposure relative to **38**. Compound **28** also exhibited an improvement in the time to reach *C*_max_ compared to **1** (15 min versus 2 h) when dosed orally (Table 8), presumably a result of its improved solubility. However, **28** was cleared quite rapidly after both oral and i.p. administration, likely due to the higher metabolic instability of the compound; it would be unlikely to deliver sufficient duration of efficacious free drug concentration at a tolerated dose. We decided to investigate alternative scaffolds with the aim of improving metabolic stability and solubility.

**Table 8:** PK data for compounds 1, 28, and 38

ID	Route	Dose [mg kg^−1^]	*C*_max_ [ng mL^−1^]^[a]^	*t*_max_ [h]^[b]^	AUC_(0–8)_ [ng min mL^−1^]^[c]^	*t*_1/2_ [h]^[d]^
**1**	p.o.	5	110	4	36 000	3
**28**	i.p.	20	5200	0.083	230 000	1.2
**28**	p.o.	20	910	0.25	69 000	1.7
**38**	i.p.	20	83	2	22 000	2.2

[a] Maximum concentration reached. [b] Time after initial dose at which *C*_max_ was reached. [c] Area under the curve. [d] Half-life of compound in the blood.

### Scaffold hopping: the core group

**Scheme 4:**
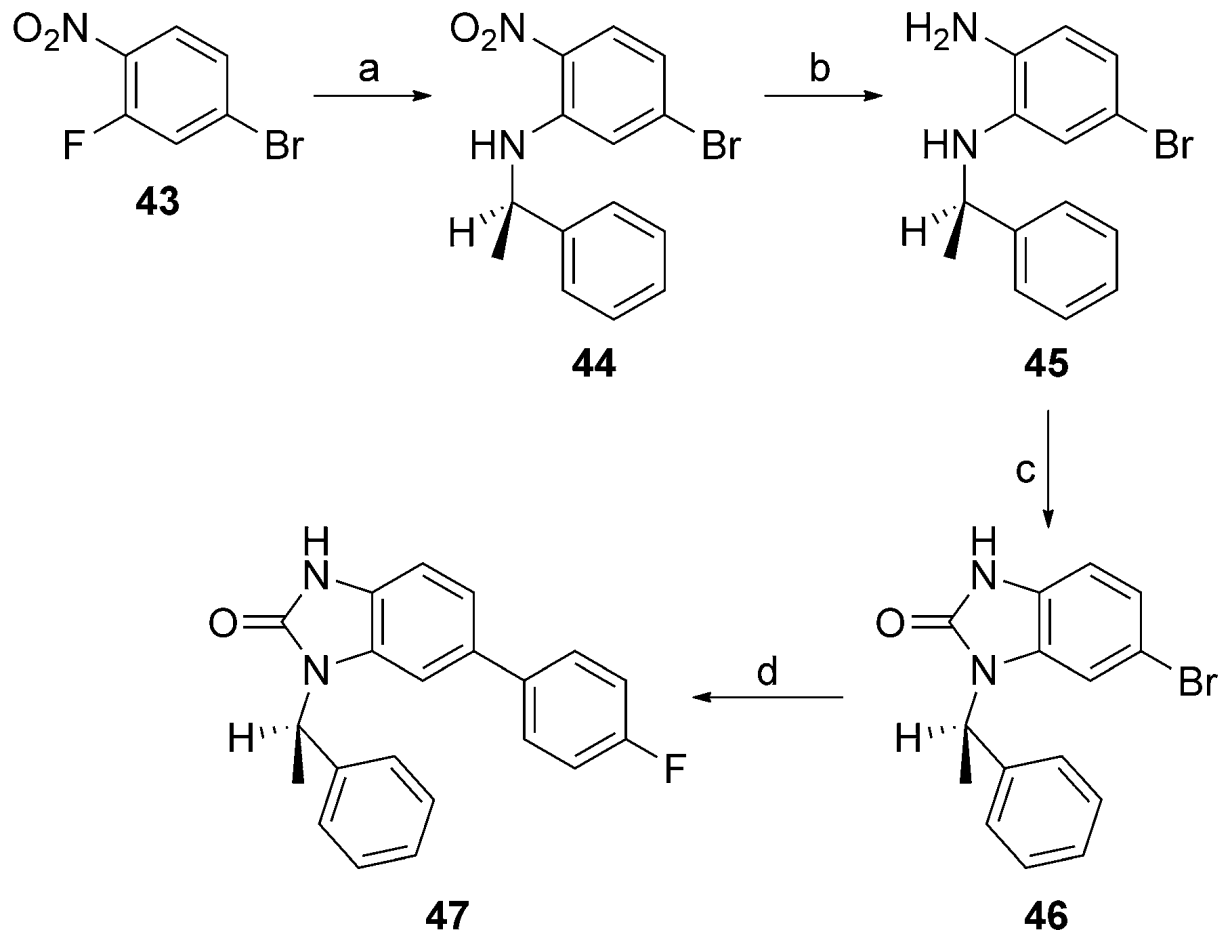
*Reagents and conditions*: a) (*R*)-methylbenzylamine, Cs_2_CO_3_, DMF, 120 °C; b) charcoal (200 % *w*/*w*), NaBH_4_, THF, H_2_O, RT, 16 h, 34 % over two steps; c) CDI, 1,4-dioxane, 140 °C, 20 min, 61 %; d) Pd(PPh_3_)_4_ (5 mol %), K_2_CO_3_ (1.5 m aqueous), boronic acid, DMF, 140 °C, 5 min, 44 %.

Initial work was conducted to investigate the importance of the pyrazine nitrogen atoms (by removal of both of them, Scheme 4). Thus, 1-bromo-3-fluoro-4-nitrophenyl **43** was reacted with α-methylbenzyl amine to give intermediate **44** (Scheme 4). The nitro group was reduced, then **45** was cyclised with CDI to give (*R*)-6-bromo-1-(1-phenylethyl)-1*H*-benzo[*d*]imidazol-2(3*H*)-one (**46**). Finally a cross-coupling reaction gave the desired compound **47**. Removal of both ring nitrogen atoms of **1** (EC_50_: 0.08 μm) to give **47** (EC_50_: 10 μm) resulted in a 100-fold loss of activity, indicating that one or both of the pyrazine nitrogen atoms are important.

**Scheme 5:**
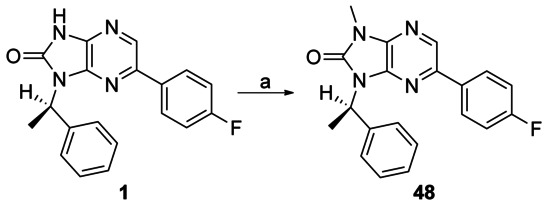
*Reagents and conditions*: a) NaH, MeI, DMF, RT, 3 h, 38 %.

**Scheme 6:**
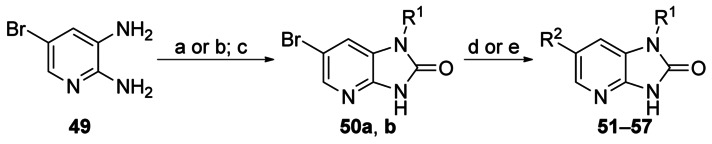
*Reagents and conditions*: a) 1) cyclohexanone, AcOH, CH_2_Cl_2_; 2) Na(OAc)_3_BH, 63 %; b) PhC(CH_3_)_2_Cl, Et_3_N, THF, 9 %; c) CDI, MeCN, 69–90 %; d) ArB(OR)_2_, K_3_PO_4_, Pd(PPh_3_)_4_, DMF/H_2_O (3:1), 130 °C (microwave), 3–69 % (compounds 51–55); e) (*S*)-PHOS, *t*BuONa, (R^2^)_2_NH, Pd_2_dba_3_, toluene, 120 °C (microwave), 32–47 % (compounds 56–57).

Capping the imidazo NH group of **1** with a methyl group gave **48**, leading to a 100-fold loss of activity (EC_50_: 8 μm versus 0.08 μm for **48** and **1**, respectively), suggesting the importance of the imidazole NH (Scheme 5). To probe the effect of the N7 nitrogen, a series of compounds were made in which the N7 nitrogen atom was replaced with CH, along with a variety of substituents at the N1- and 6-positions. 2,3-Diamino-5-bromopyridine **49** was subjected to either reductive amination or alkylation conditions to introduce the N3 substituent, followed by cyclisation with CDI to afford the key 6-bromo- 1-(*R*)-1*H*-imidazo[4,5-*b*]pyrazin-2(3*H*)-one scaffold **50 a**–**b** (Scheme 6). Treatment of this under either Suzuki or Buchwald conditions afforded desired 6-substituted analogues (**51**–**57**).

Removal of N7 was tolerated if R^1^ was retained as cyclohexyl (**51**) and R^2^ was retained as a phenyl ring (Table 9). Replacement of the phenyl group at R^2^ with a piperidine (**56**) was also tolerated. However, introduction of either 3-pyridyl- (**55**), morpholino- (**57**), or (2-hydroxymethyl)phenyl groups (**53**) gave rise to compounds that were approximately 2-, 7- and 13-fold less active than their N7 homologues (**30**, **32**, and **28**, respectively). At the R^1^ position, replacement with 2-phenylpropan-2-yl groups (**52**, **54**) was less favourable.

**Table 9:** Replacement of N7 nitrogen

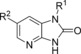						
ID	R^1^	R^2^	clog*P*	EC_50_ [μm]^[a]^		Aq. Sol.
				*T. b. brucei*	MRC5	[μm]^[b]^
**51**	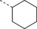	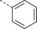	3.9	0.054	>50	36
**52**	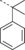	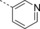	3.1	0.79	23	ND
**53**	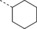	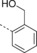	2.8	0.67	>50	ND
**54**	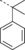	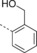	3.2	15	>50	ND
**55**	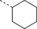	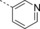	2.8	0.23	>50	180
**56**	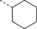	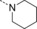	3.3	0.093	>50	>250
**57**	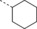	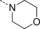	2.2	0.33	>50	ND

[a] Values are the geometric mean of two or more determinations; standard deviation is typically within 2-fold of the EC_50_ value. [b] Aqueous kinetic solubility. ND: not determined.

It was hypothesised that the “urea” moiety may be a cause of the poor solubility, possibly through its ability to cause antiparallel hydrogen bonded dimers. However, if this was to be replaced, certain features would need to be retained: the carbonyl group could act as a hydrogen bond acceptor, and the NH group as a hydrogen bond donor. As N1 was a tertiary nitrogen it was hypothesised that does not make any important interactions and could be replaced by a carbon atom. This would then allow the carbonyl to be replaced by a nitrogen, which could also act as a hydrogen bond acceptor, giving an aza-indazole scaffold: a known kinase scaffold.

**Scheme 7:**
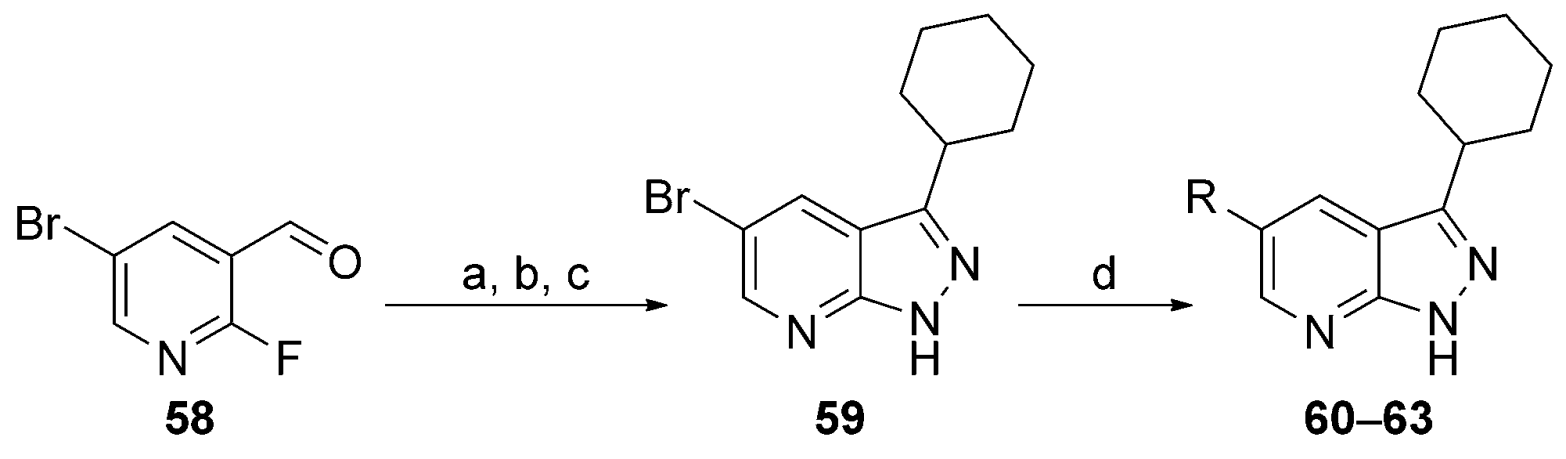
*Reagents and conditions*: a) cyclohexylmagnesium bromide, THF, −78 °C→RT, 55 %; b) PDC, CH_2_Cl_2_, 0 °C→RT, 91 %; c) NH_2_NH_2_⋅H_2_O, EtOH, reflux, 95 %; d) RB(OH)_2_, 2 m Na_2_CO_3_, Pd(PPh_3_)_4_, DME/H_2_O/EtOH (7:3:2), 140 °C, microwave, 16–69 %.

The key intermediate 5-bromo-3-cyclohexyl-1*H*-pyrazolo[3,4-*b*]pyridine **59** was synthesised in three steps from commercially available 5-bromo-2-fluoronicotinaldehyde **58** and was subsequently reacted under Suzuki conditions to give the corresponding 5-aryl-/heteroaryl-substituted compounds **60**–**63** (Scheme 7). Both phenyl (**60**) and 3-pyridyl (**63**) were tolerated, with the former showing similar levels of potency and selectivity to the other core scaffolds bearing an unsubstituted phenyl group (**51** EC_50_=0.054 μm and **19** EC_50_=0.04 μm; Table 10). Phenyl groups (**61** and **62**) bearing a substituent at the 2-position were less favourable.

**Table 10:** SAR for aza-indazoles

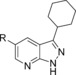					
ID	R	clog*P*	EC_50_ [μm]^[a]^		Aq. Sol.
			*T. b. brucei*	MRC5	[μm]^[b]^
**60**	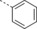	4.7	0.059	>50	20
**61**	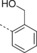	3.6	1.3	>50	ND
**62**	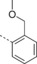	4.7	2.6	>50	ND
**63**	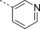	3.5	0.26	>50	250

[a] Values are the geometric mean of two or more determinations; standard deviation is typically within 2-fold of the EC_50_ value. [b] Aqueous kinetic solubility. ND: not determined.

Removal of one the nitrogen atoms from the pyrazine ring was predicted to lead to improvements in metabolic stability, protein binding, and solubility (Table 11). However, replacement of the core scaffold with the aza-indazole had a negative effect on the in vitro DMPK properties of the compounds (compare **51** and **60**; **55** and **63**).

**Table 11:** In vitro ADMET data

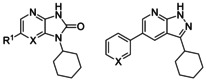								
ID	R^1^	X	EC_50_ [μm]^[a]^		*CL*_int_	PPB	Aq. Sol.	tPSA
			*T. b. brucei*	MRC5	[mL min^−1^ g^−1^]	[% free]	[μm]^[b]^	[Å^2^]
**28**	(2-CH_2_OH)Ph	N	0.046	>50	2.4	7.2	>250	83
**19**	Ph	N	0.044	>50	3.6	0.8	ND	64
**51**	Ph	CH	0.054	>50	5.8	2.9	36	51
**30**	3-pyridyl	N	0.12	>50	2.8	2.3	ND	76
**55**	3-pyridyl	CH	0.23	>50	2.7	11	180	64
**31**	piperidine	N	0.077	>50	15	0	ND	67
**56**	piperidine	CH	0.093	>50	3.6	11	>250	54
**60**	–	CH	0.059	>50	15	1.4	20	42
**63**	–	N	0.26	>50	6.6	5.2	250	54

[a] Values are the geometric mean of two or more determinations; standard deviation is typically within 2-fold of the EC_50_ value. [b] Aqueous kinetic solubility. ND: not determined.

Compound **56** was selected for in vivo PK studies, as it had the most favourable in vitro profile (potency, metabolic stability, protein binding, and solubility). However, **56** had lower exposure levels than **28** and was also cleared rapidly, in line with its higher *CL*_int_ value (Table 12).

**Table 12:** PK data for compounds 28 and 56

ID	Route	Dose [mg kg^−1^]	*C*_max_ [ng mL^−1^]^[a]^	*t*_max_ [h]^[b]^	AUC_(0–8)_ [ng min mL^−1^]^[c]^	*t*_1/2_ [h]^[d]^
**28**	i.p.	20	5200	0.083	230 000	1.2
**56**	i.p.	10	580	0.25	53 000	0.7

[a] Maximum concentration reached. [b] Time after initial dose at which *C*_max_ was reached. [c] Area under the curve. [d] Half-life of compound in the blood.

### Determination of the mode of the antiparasitic effect: static or cidal action

**Figure 1:**
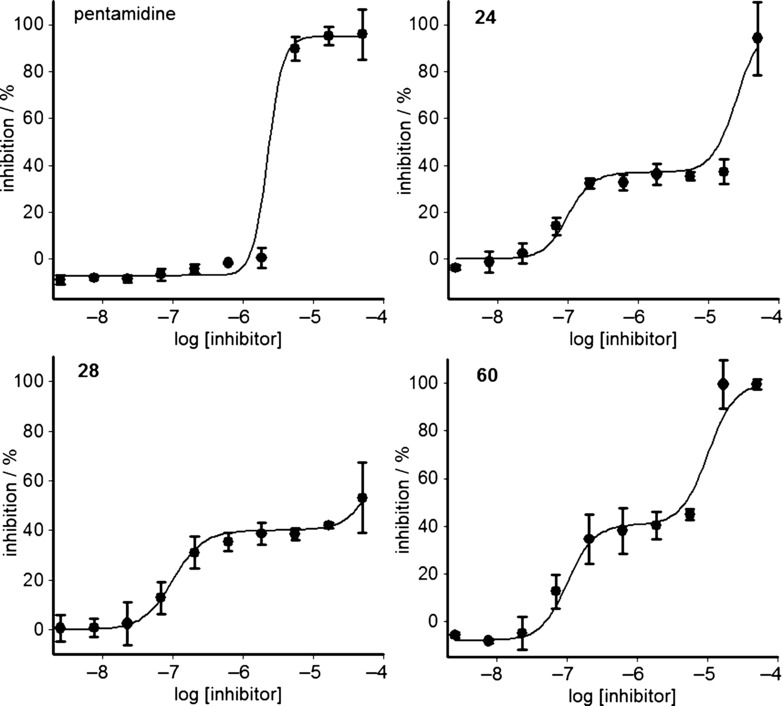
Static/cidal dose–response curves for pentamidine, 24, 28, and 60. Data are the average of triplicate measurements after 48 h incubation of *T. b. brucei* cells with a) pentamidine (cidal), b) compound 24 (static at concentration tested), c) compound 28 (growth-slowing), and d) compound 60 (cidal). Inhibitors were tested in a 10-point dose–response curve at a 1:3 dilution factor; data were analysed with either a monophasic or a biphasic fit. Static and cidal are defined by analysis of the growth curves as shown in the Supporting Information (Figure S4), where cidal compounds show a decrease in the numbers of parasites.

During the course of these studies, work from parallel series indicated that compounds that are cytostatic against *T. brucei* are not effective in animal models of infection. Therefore compounds that only exert a static effect (i.e., stopping the growth of cells, whilst not killing them) or a decrease in growth rate are unlikely to clear the parasites completely and should be removed from drug discovery pipelines as early as possible. To provide information regarding the mode of action of the compounds discussed, we selected a number of examples to run in a static–cidal assay.[22,26] Compounds with either an imidazo[4,5-*b*]pyrazin-2(3*H*)-one (**24, 26, 28, 30**, and **39**) or imidazo[4,5-*b*]pyridin-2(3*H*)-one (**51**, **55**, and **56**) core showed growth-slowing or static behaviour at all concentrations tested, whereas compounds containing a 1*H*-pyrazolo[3,4-*b*]pyridine core (**60** and **63**) showed cidal behaviour at a minimum cidal concentration of 17 μm (Table 13). With the exception of pentamidine and melarsoprol, all compounds showed either biphasic dose–response curves or partial curves. This suggested these compounds may act on at least two targets, the first of which caused a growth-slowing or static mode of action, whilst inhibition of the subsequent target(s) had a cytocidal effect (Figure 1). Only pentamidine, melarsoprol, **60**, and **63** reached a well-defined plateau at 100 % inhibition, which explains why only these compounds showed clear cidal activity.

**Table 13:** Static/cidal data

ID	*T. b. brucei* EC_50_ [μm]^[a]^	MCC [μm]^[b]^	SC EC_50_(1) [μm]^[b]^	SC EC_50_(2) [μm]^[b]^	Mode^[c]^
**24**	0.03	>50	0.07	30	S
**26**	0.06	>50	0.09	–	GS
**28**	0.05	>50	0.10	>50	GS
**30**	0.12	>50	0.23	–	GS
**39**	0.20	>50	0.41	32	S
**51**	0.05	>50	0.02	41	S^[d]^
**55**	0.23	>50	0.33	–	GS
**56**	0.09	>50	0.07	>50	GS
**60**	0.06	17	0.08	7.9	C
**63**	0.26	17	0.28	6.1	C
pentamidine	0.004	0.1	0.019	–	C
melarsoprol	0.009	0.1	0.031	–	C

[a] Values determined by the standard *T. brucei* cell viability assay and are the geometric mean of two or more determinations; standard deviation is typically within 2-fold of the EC_50_ value. [b] Compounds were also tested in the static–cidal assay carried out in triplicate to determine minimal cidal concentration (MCC) and static cidal EC_50_ values for the first [SC EC_50_(1)] and second [SC EC_50_(2)] sites. EC_50_ and SC EC_50_(1) are expected to represent the same mode of action of the compounds and are therefore very similar.[26] [c] GS: growth-slowing, S: static, C: cidal. [d] Indication of cidality at 50 μm.

The mode(s) of action of these compounds are not known. Analysis of the literature suggests that these chemotypes have been investigated against many families of enzyme in humans, including protein kinases,[27–32] hydrolases,[33] and transferases.[34] Our work described in Figure 1 suggests that there are likely to be several modes of action in trypanosomes.

Given our experience from other programmes, in order for a compound to show efficacy in a mouse model of HAT, it is important that the compound is cidal, rather than static, in its activity against *T. brucei*. Compounds that are consistent with this are **60** and **63**. However, given their metabolic instability and poor potency (minimum cidal concentrations of 17 μm), it is highly unlikely that either would deliver efficacy in a mouse model of HAT. Further work is needed to improve the potency and pharmacokinetic properties of the molecules.

## Conclusions

Through our phenotypic screening approach with a relatively small library, we have developed a series of compounds that exhibit good levels of potency in the *T. b. brucei* growth inhibition assay, and that are nontoxic to MRC5 mammalian cells. Despite the fact that the screening library was a focused protein kinase library, it was possible to obtain compounds with selectivity over the mammalian protein kinases assayed. These compounds possess reasonable physicochemical properties and show oral exposure, but have modest half-lives. By changing the substituents R^1^ and R^2^ it was possible to optimise the compounds for potency and to alter key physicochemical and DMPK properties, in the absence of knowledge of the molecular target. It was also possible to scaffold-hop, although it is possible that the new scaffold works on a different molecular target.

The ultimate aim would be to devise a compound that has oral bioavailability and the capacity to penetrate the BBB. The compounds prepared in this study generally have physicochemical properties consistent with BBB penetration as reported by Wager,[19] in terms of *M*_r_, tPSA, clog*P*, and number of hydrogen bond donors. Although of course for compounds taken forward, BBB permeability would have to be determined experimentally.

Key compounds in a static–cidal assay showed an unusual biphasic nature, giving two points of inflection on the dose–response curve. This suggests at least two modes of action (or, less likely due to the use of a clonal line, differential susceptibility of cell sub-populations). Compounds from the 1*H*-imidazo[4,5-*b*]pyrazin-2(3*H*)-one scaffold appeared to be either static or growth-slowing, whilst compounds from the 1*H*-pyrazolo[3,4-*b*]pyridine series were cidal. It is possible that compounds from the 1*H*-imidazo[4,5-*b*]pyrazin-2(3*H*)-one scaffold will be cidal at higher concentrations, and that the key difference between the two scaffolds is not a difference in mode-of-action but a difference in potency against the target(s) represented by the second phase of the biphasic curves.

A way forward would be to identify the molecular target(s) of the compounds, specifically which target(s) causes the cytocidal activity, and to focus efforts on potent inhibition of this target. Subsequent progression of the series will require identification of compounds that are cytocidal. It will also be necessary to optimise the key properties of the molecule (microsomal stability, solubility, and protein binding). Although it has proved possible to optimise the compound in terms of potency and to some extent at least the pharmacokinetic parameters, we have reached a biological issue which has proved difficult to resolve in the absence of knowledge of the cidal target. In this case, a combination of phenotypic and target-based approaches would probably be optimal.

## Experimental Section

### Chemistry

Compounds **1**, **2, 3** and **13** were purchased from BioFocus. Chemicals and solvents were purchased from Aldrich Chemical Co., Fluka, ABCR, VWR, Acros, Fisher Chemicals, and Alfa Aesar and were used as received unless otherwise stated. Air- and moisture-sensitive reactions were carried out under an inert atmosphere of argon in oven-dried glassware. Analytical thin-layer chromatography (TLC) was performed on pre-coated TLC plates (layer 0.20 mm silica gel 60 with fluorescent indicator UV254, Merck). Developed plates were air dried and analyzed under a UV lamp (*λ* 254/365 nm). Flash column chromatography was performed using prepacked silica gel cartridges (230–400 mesh, 40–63 μm, SiliCycle) (unless otherwise stated) using a Teledyne ISCO Combiflash Companion or Combiflash Retrieve. ^1^H and ^13^C NMR spectra were recorded on a Bruker Avance II 500 spectrometer (^1^H at 500.1 MHz, ^13^C at 125.8 MHz), or a Bruker DPX300 spectrometer (^1^H at 300.1 MHz). Chemical shifts (*δ*) are expressed in ppm recorded using the residual solvent as internal reference in all cases. Signal splitting patterns are described as singlet (s), doublet (d), triplet (t), quartet (q), pentet (p), multiplet (m), broad (br), or a combination thereof. Coupling constants (*J*) are quoted to the nearest 0.1 Hz. LC–MS analyses were performed with either an Agilent HPLC 1100 series instrument connected to a Bruker Daltonics MicrOTOF or an Agilent Technologies 1200 series HPLC connected to an Agilent Technologies 6130 quadrupole spectrometer, where both instruments were connected to an Agilent diode array detector. LC–MS chromatographic separations were conducted with a Waters Xbridge C_18_ column, 50 mm×2.1 mm, 3.5 μm particle size; mobile phase: H_2_O/MeCN+0.1 % HCOOH, or H_2_O/MeCN+0.1 % NH_3_; linear gradient from 80:20 to 5:95 over 3.5 min and then held for 1.5 min; flow rate: 0.5 mL min^−1^. All tested compounds had a measured purity of ≥95 % (by TIC [total ion current] and UV) as determined by this analytical LC–MS system. High-resolution electrospray MS measurements were performed on a Bruker Daltonics MicrOTOF mass spectrometer. Microwave-assisted chemistry was performed using a Biotage initiator microwave synthesiser.

**1-Benzyl-6-(4-fluorophenyl)-1*H*-imidazo[4,5-*b*]pyrazin-2(3*H*)-one (9)**: A mixture of 3,5-dibromopyrazin-2-amine (400 mg, 1.58 mmol), benzylamine (189 μL, 1.74 mmol) and DIPEA (302 μL, 1.74 mmol) in EtOH (2 mL) was heated under microwave irradiation at 180 °C for 90 min. The reaction was cooled to room temperature and concentrated in vacuo. The resulting slurry was partitioned between CH_2_Cl_2_ (20 mL) and H_2_O (10 mL), the organic layer was washed with brine (10 mL) and concentrated in vacuo. The resultant crude residue was purified by column chromatography, eluting with 20 % EtOAc/hexane to give N2-benzyl-6-bromopyrazine-2,3-diamine (**6 c**; 281 mg, 64 %) as a brown oil. Compound **6 c** (281 mg, 1.10 mmol), 4-fluorophenylboronic acid (154 mg, 1.10 mmol), tetrakis(triphenylphosphine)palladium(0) (58 mg, 0.05 mmol), potassium carbonate (414 mg, 3.0 mmol) in H_2_O (5 mL), and DMF (5 mL) were combined and heated under microwave irradiation at 160 °C for 5 min. The reaction was then cooled to room temperature, and then diluted with a 10:1 mixture of CH_2_Cl_2_/MeOH (10 mL). The mixture was poured onto an SCX-2 column and allowed to drip through; it was then washed with a 10:1 mixture of CH_2_Cl_2_/MeOH (10 mL). The SCX-2 column was then eluted with NH_3_ in MeOH (7 n, 20 mL) and the eluates were concentrated in vacuo. The resultant brown solid was dissolved in 1,4-dioxane (2 mL), and CDI (140 mg, 0.86 mmol) was added. The mixture was heated under microwave irradiation at 140 °C for 20 min, cooled to room temperature and concentrated in vacuo. The resultant crude residue was purified by column chromatography, eluting with 0–100 % EtOAc/petroleum ether (PE) 40–60 °C to give the title compound **9** (47 mg, 15 %) as an off-white solid. ^1^H NMR (500 MHz, [D_6_]DMSO): *δ*=12.19 (s, 1 H), 8.53 (s, 1 H), 8.09–8.06 (br s, 2 H), 7.43–7.27 (m, 7 H), 5.08 ppm (s, 2 H); LRMS (ES+): *m*/*z* (%) 321.0 [*M*+H]^+^ (100); HRMS (*m*/*z*): [*M*+H]^+^ calcd for C_18_H_14_FN_4_O: 321.1146, found: 321.1131.

**(*R*)-6-Phenyl-1-(1-phenylethyl)-1*H*-imidazo[4,5-*b*]pyrazin-2(3*H*)-one (14)**: (*R*)-6-bromo-*N*^2^-(1-phenylethyl)pyrazine-2,3-diamine (**6 a**; 100 mg, 0.35 mmol), phenyl boronic acid (44 mg, 0.35 mmol), tetrakis(triphenylphosphine)palladium(0) (20 mg, 0.018 mmol), potassium carbonate (700 μL of a 1.5 m solution in H_2_O, 1.05 mmol), and DMF (2 mL) were combined and heated under microwave irradiation at 160 °C for 5 min. The reaction was then cooled to room temperature, and then diluted with a 10:1 mixture of CH_2_Cl_2_/MeOH (10 mL). The mixture was poured onto an SCX-2 column and allowed to drip through, then washed with a 10:1 mixture of CH_2_Cl_2_/MeOH (10 mL). The SCX-2 column was then eluted with NH_3_ in MeOH (7 n, 20 mL), and the eluates were concentrated in vacuo. The resultant brown solid was dissolved in 1,4-dioxane (2 mL), and CDI (140 mg, 0.86 mmol) was added. The mixture was heated under microwave irradiation at 140 °C for 20 min, cooled to room temperature and concentrated in vacuo. The resultant crude residue was purified by column chromatography, eluting with 0–100 % EtOAc/PE 40–60 °C to give the title compound **14** (21 mg, 18 %) as a pale-yellow solid. ^1^H NMR (500 MHz, [D_6_]DMSO): *δ*=12.12 (s, 1 H), 8.51 (s, 1 H), 8.00 (d, *J*=7.3 Hz, 2 H), 7.55–7.47 (m, 4 H), 7.42–7.34 (m, 3 H), 7.27 (t, *J*=7.3 Hz, 1 H), 5.74 (q, *J*=7.2 Hz, 1 H), 2.03 ppm (d, *J*=7.3 Hz, 3 H); LRMS (ES+): *m*/*z* (%) 317.1 [*M*+H]^+^ (97); HRMS (*m*/*z*): [*M*+H]^+^ calcd for C_19_H_17_N_4_O: 317.1397, found: 317.1394.

**1-Cyclohexyl-6-(4-fluorophenyl)-1*H*-imidazo[4,5-*b*]pyrazin-2(3*H*)-one (12)**: 6-Bromo-1-cyclohexyl-1*H*-imidazo[4,5-*b*]pyrazin-2(3*H*)-one **7 b** (100 mg, 0.33 mmol), tetrakis(triphenylphosphine)palladium(0) (12 mg, 0.01 mmol), potassium carbonate (0.65 mL of a 1.5 m solution in H_2_O, 1.0 mmol), 4-fluorophenyl boronic acid (46 mg, 0.33 mmol), in DMF (2.0 mL) were combined and the mixture was heated under microwave irradiation at 180 °C for 5 min. The reaction mixture was absorbed onto silica and purified by chromatography eluting with 50 % EtOAc/PE 40–60 °C to give the title compound **12** (17 mg, 16 %) as a yellow solid. ^1^H NMR (500 MHz, [D_6_]DMSO): *δ*=12.04 (s, 1 H), 8.49 (s, 1 H), 8.10–8.07 (m, 2 H), 7.34 (t, *J*=8.8 *Hz*, 2 H), 4.28–4.22 (m, 1 H), 2.37–2.29 (m, 2 H), 1.88–1.80 (m, 4 H), 1.71 (d, *J*=12.8 Hz, 1 H), 1.44–1.36 (m, 2 H), 1.29–1.22 ppm (m, 1 H); LRMS (ES+): *m*/*z* (%) 313.2 [*M*+H]^+^ (100); HRMS (*m*/*z*): [*M*+H]^+^ calcd for C_17_H_18_FN_4_O: 313.1459, found: 313.1458.

**1-Cyclohexyl-6-(piperidin-1-yl)-1*H*-imidazo[4,5-*b*]pyrazin-2(3*H*)-one (31)**: 6-Bromo-1-cyclohexyl-1*H*-imidazo[4,5-*b*]pyrazin-2(3*H*)-one (100 mg, 0.33 mmol), tris(dibenzylideneacetone)dipalladium (16 mg, 0.017 mmol), potassium *tert*-butoxide (114 mg, 1.0 mmol), (±)-2,2′-bis(diphenylphosphino)-1,1′-binaphthyl (22 mg, 0.034 mmol), piperidine (67 μL, 0.68 mmol) and 1,4-dioxane (2.0 mL) were combined and heated under microwave irradiation at 180 °C for 5 min. The reaction was cooled to room temperature, absorbed onto silica and purified by column chromatography, eluting with 50 % EtOAc/hexane to give the title compound **31** (25 mg, 24 %) as a yellow solid. ^1^H NMR (500 MHz, [D_6_]DMSO): *δ*=11.36 (s, 1 H), 7.45 (s, 1 H), 4.16–4.09 (m, 1 H), 3.38–3.32 (m, 2 H), 2.52–2.50 (m, 2 H), 2.27–2.19 (m, 2 H), 1.83 (d, *J*=13.0 Hz, 2 H), 1.73–1.67 (m, 3 H), 1.59 (br s, 6 H), 1.39–1.31 (m, 2 H), 1.21–1.13 ppm (m, 1 H); LRMS (ES+): *m*/*z* (%) 302.2 [*M*+H]^+^ (100); HRMS (*m*/*z*): [*M*+H]^+^ calcd for C_16_H_24_N_5_O: 302.1975, found: 302.1973.

**1-(1-Methoxypropan-2-yl)-6-phenyl-1*H*-imidazo[4,5-*b*]pyrazin-2(3*H*)-one (39)**: 3-Bromo-5-phenylpyrazine-2-amine (125 mg, 0.5 mmol), 1-methoxybutan-2-amine (77 mg, 0.75 mmol), DIPEA (0.1 mL), and *n*BuOH (0.5 mL) were heated under microwave irradiation at 220 °C for 1 h. The reaction was diluted with EtOAc (10 mL), washed with H_2_0 (3×10 mL), the organic layer dried over MgSO_4_, filtered, and the solvent was removed in vacuo. Column chromatography eluting with CH_2_Cl_2_ to CH_2_Cl_2_/MeOH (95:5) afforded the desired substituted 6-phenylpyrazine-2,3-diamine, which was used for subsequent reaction without further purification. Substituted 6-phenylpyrazine-2,3-diamine (0.5 mmol), CDI (97 mg, 0.6 mmol), and 1,4-dioxane (1 mL) were heated under microwave irradiation at 130 °C for 10 min. The crude reaction mixture was partitioned between EtOAc and H_2_O, the organic layer was dried over MgSO_4_, filtered, and the solvent removed in vacuo. The resultant crude residue was purified by column chromatography, eluting with EtOAc to give the title compound **39** (13 mg, 10 %) as a colourless solid. ^1^H NMR (500 MHz, CDCl_3_): *δ*=8.90 (br s, 1 H), 8.41 (s, 1 H), 7.99–7.97 (m, 2 H), 7.51 (t, *J*=7.3 Hz, 2 H), 7.44 (t, *J*=7.3 Hz, 1 H), 4.98–4.94 (m, 1 H), 4.27 (t, *J*=10.1 Hz, 1 H), 3.68 (dd, *J*=10.1, 5.2 Hz, 1 H), 3.37 (s, 3 H), 1.65 ppm (d, *J*=7.1 Hz, 3 H); LRMS (ES+): *m*/*z* (%) 285.1 [*M*+H]^+^ (100); HRMS (*m*/*z*): [*M*+H]^+^ calcd for C_15_H_16_N_4_O_2_: 285.1346; found, 285.1336.

### Biological assays

*Medium-throughput screen for African trypanosomes and cytotoxicity*: Measurement of inhibition of the proliferation of L-6 cells (mouse muscle fibroblasts) and *T. brucei* bloodstream-stage cells was performed as previously described.[35,36]

*Trypanosome and MRC5 proliferation assay*: Measurement of inhibition of the proliferation of MRC5 cells (human lung fibroblasts) and *T. brucei* bloodstream-stage cells was performed using a modification of a cell viability assay previously described.[35,36] Compounds (50 mm to 0.5 nm) were incubated with 2×10^3^ cells per well in 0.2 mL of the appropriate culture medium (MEM with 10 % foetal bovine serum for MRC5 cells) in clear 96-well plates. Plates were incubated at 37 °C in the presence of 5 % CO_2_ for 69 h. Resazurin was then added to a final concentration of 50 μm, and plates were incubated as above for a further 4 h before being read on a BioTek Flx800 fluorescence plate reader.

*Determination of cidality* against *T. brucei* bloodstream-stage cells was performed using a static–cidal (SC) assay as previously reported.[26] Briefly, bloodstream-form trypanosomes were seeded into 384-well plates at 4×10^5^ mL^−1^ (50 μL per well), followed by immediate addition of resazurin (50 μm final) to one of the plates, and all plates were incubated at 37 °C, 5 % CO_2_. After 4 h the *t*_0_ plate was read using a PerkinElmer Victor 3 plate reader (*λ*_ex_=528 nm, *λ*_em_=590 nm). The second plate was processed in the same manner 20 h later, and at 44 h the last plate was processed. The minimum cidal concentration (MCC) is defined as the lowest concentration of drug that results in a decrease of resorufin signal over time. For dose–response curves from this assay either a monophasic or a biphasic equation was used, depending on which one provided the best fit. For monophasic fits, the following four-parametric equation [Eq. (1)] was used:


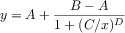
(1)

in which *A*=percent inhibition at bottom, *B*=percent inhibition at top, *C*=EC_50_, *D*=slope, *x*=inhibitor concentration, and *y*=percent inhibition. Equation (2) was used for biphasic fits:


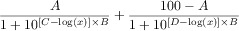
(2)

for which *A*=percent inhibition at mid-plateau, *B*=slope, *C*=log[SC EC_50_(1)], *D*=log[SC EC_50_(2)]. Thus SC EC_50_(1) is the EC_50_ for the first phase of the curve, and SC EC_50_(2) is that for the second phase. Inhibition at the bottom of the curve is fixed at 0 % and at the top, 100 %.

*Intrinsic clearance experiments*: Test compound (0.5 μm) was incubated with female CD1 mouse liver microsomes (Xenotech LLC; 0.5 mg mL^−1^ 50 mm potassium phosphate buffer, pH 7.4), and the reaction started with the addition of excess NADPH (8 mg mL^−1^ 50 mm potassium phosphate buffer, pH 7.4). Immediately, at *t*_0_, then at 3, 6, 9, 15, and 30 min an aliquot (50 μL) of the incubation mixture was removed and mixed with MeCN (100 μL) to stop the reaction. Internal standard was added to all samples, the samples were centrifuged to sediment precipitated protein, and the plates were then sealed prior to UPLC–MS/MS analysis using a Quattro Premier XE instrument (Waters Corp., USA). XLfit (IDBS, UK) was used to calculate the exponential decay and consequently the rate constant (*k*) from the ratio of peak area of test compound to internal standard at each time point. The rate of intrinsic clearance [*CL*_int_, mL min^−1^ (g liver)^−1^] of each test compound was then calculated with Equation (3):


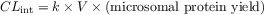
(3)

in which *V* [mL (mg protein)^−1^] is the incubation volume per mg protein added, and microsomal protein yield is taken as 52.5 mg protein per gram of liver. Verapamil (0.5 μm) was used as a positive control to confirm acceptable assay performance.

*Plasma protein binding experiments*: In brief, a 96-well equilibrium dialysis apparatus was used to determine the free fraction in plasma for each compound (HT Dialysis LLC, Gales Ferry, CT, USA). Membranes (12–14 kDa cutoff) were conditioned in deionised H_2_O for 60 min, followed by conditioning in 80:20 deionised H_2_O/EtOH for 20 min, and then rinsed in isotonic buffer before use. Female CD1 mouse plasma was removed from the freezer and allowed to thaw on the day of experiment. Thawed plasma was then centrifuged (Allegra X12-R, Beckman Coulter, USA), spiked with test compound (10 μg g^−1^), and 150 μL aliquots (*n*=6 replicate determinations) loaded into the 96-well equilibrium dialysis plate. Dialysis against isotonic buffer (150 μL) was carried out for 5 h in a temperature-controlled incubator at ∼37 °C (Barworld Scientific Ltd., UK) using an orbital microplate shaker at 125 rpm (Barworld Scientific). At the end of the incubation period, aliquots of plasma or buffer were transferred to micronic tubes (Micronic B.V., the Netherlands), and the composition in each tube was balanced with control fluid such that the volume of buffer to plasma is the same. Sample extraction was performed by the addition of 400 μL MeCN containing an appropriate internal standard. Samples were allowed to mix for 1 min and then centrifuged at 3000 rpm in 96-well blocks for 15 min (Allegra X12-R, Beckman Coulter, USA). All samples were analysed by UPLC–MS/MS on a Quattro Premier XE Mass Spectrometer (Waters Corp.). The unbound fraction was determined as the ratio of the peak area in buffer to that in plasma.

*Solubility experiments*: The kinetic aqueous solubility of the test compounds was measured using laser nephelometry. Compounds were subject to serial dilution from 10 mm to 0.5 mm in DMSO. An aliquot was then mixed with Milli-Q H_2_O to obtain an aqueous dilution plate with a final concentration range of 250–12 μm, with a final DMSO concentration of 2.5 %. Triplicate aliquots were transferred to a flat-bottomed polystyrene plate which was immediately read on the NEPHELOstar (BMG Lab Technologies). The amount of laser scatter caused by insoluble particulates (relative nephelometry units, RNU) was plotted against compound concentration using a segmental regression fit, with the point of inflection being quoted as the compounds′ aqueous solubility (in μm).

*In vivo pharmacokinetics in mice*: Compounds **1** and **28** were dosed orally by gavage as a solution at 5 mg free base per kg or 20 mg free base per kg, respectively (dose volume: 5 or 10 mL kg^−1^, dose vehicle: 5 % DMSO, 40 % PEG400, 55 % distilled H_2_O) to female NMRI mice (*n*=3). Compounds **28**, **38**, and **56** were dosed as a bolus solution intraperitoneally at 10 (**56**) or 20 (**28** and **38**) mg free base per kg (dose volume: 10 mL kg^−1^, dose vehicle: 5 % DMSO, 40 % PEG400, 55 % sterile H_2_O) to female NMRI mice (*n*=3). Female NMRI mice were chosen, as these represent the sex and strain used for the stage-1 and stage-2 HAT efficacy models. Blood samples were taken from each mouse at 5 (i.p. only), 15 and 30 min, 1, 2, 4, 6 and 8 h post-dose and mixed with two volumes of distilled H_2_O. After suitable sample preparation, the concentration of each compound in blood was determined by UPLC–MS/MS using a Quattro Premier XE (Waters, USA). Pharmacokinetic parameters were derived from the mean blood concentration time curve using PKsolutions software ver. 2.0 (Summit Research Services, USA).

*Ethics*: All regulated procedures on living animals were carried out under the authority of a project license issued by the Home Office under the Animals (Scientific Procedures) Act 1986, as amended in 2012 (and in compliance with EU Directive EU/2010/63). License applications will have been approved by the University's Ethical Review Committee (ERC) before submission to the Home Office. The ERC has a general remit to develop and oversee policy on all aspects of the use of animals on University premises and is a subcommittee of the University Court, its highest governing body.

### Abbreviations

PPB: plasma protein binding; *CL*_int_: intrinsic clearance; MCC: minimum cidal concentration; SC: static/cidal; NBS: *N*-bromosuccinimide; DIPEA: *N*,*N*-diisopropylethylamine; CDI: 1,1′-carbonyldiimidazole; DMF: *N*,*N*-dimethylformamide; THF: tetrahydrofuran; (*S*)-PHOS: 2-dicyclohexylphosphino-2′,6′-dimethoxybiphenyl; *rac*-binap: (±)-2,2′-bis(diphenylphosphino)-1,1′-binaphthyl.

## Supporting Information

As a service to our authors and readers, this journal provides supporting information supplied by the authors. Such materials are peer reviewed and may be re-organized for online delivery, but are not copy-edited or typeset. Technical support issues arising from supporting information (other than missing files) should be addressed to the authors.
