# Supplementary material for: Discovery of Inhibitors of Trypanosoma brucei by Phenotypic Screening of a Focused Protein Kinase Library
Source: ChemMedChem. 2015 Sep 18;10(11):1809–20. doi: 10.1002/cmdc.201500300 (PMC4648050; doi:10.1002/cmdc.201500300)

CHEMMEDCHEM
Chemistry & Drug Discovery

Supporting Information

Discovery of Inhibitors of *Trypanosoma brucei* by Phenotypic Screening of a Focused Protein Kinase Library

Andrew Woodland,[a] Stephen Thompson,[a] Laura A. T. Cleghorn,[a] Neil Norcross,[a] Manu De Rycker,[a] Raffaella Grimaldi,[a] Irene Hallyburton,[a] Bhavya Rao,[a] Suzanne Norval,[a] Laste Stojanovski,[a] Reto Brun,[b] Marcel Kaiser,[b] Julie A. Frearson,[a] David W. Gray,[a] Paul G. Wyatt,[a] Kevin D. Read,[a] and Ian H. Gilbert*[a]

cmdc_201500300_sm_miscellaneous_information.pdf

SUPPORTING INFORMATION

Table of contents:

1. Synthetic procedures for compounds **5-60**
2. Kinase Profiling
3. SOP for the initial screen of the DDU kinase set
4. Static/Cidal dose reponse and kill curves.

S1. **Synthetic procedures for compounds 5-60**

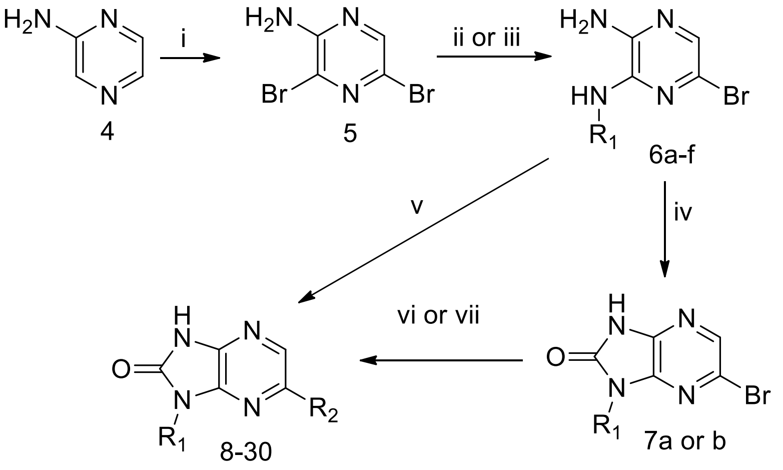

Scheme 1:

i. NBS ,MeCN, 18h, rt., 12%; ii. Amine, DIPEA, EtOH, 180 ºC, µW, 30 min, 27-64%; iii. cyclohexylamine, DIPEA, n-BuOH, 220 ºC, µW, 30 min, 95%; iv. CDI, 1,4-dioxane, 80 ºC, 16 h, 31% ; v. For compounds **8-11, 13-15** and **17-18; a) $Pd(PPh_3)_4$ (3 mol%), K_2CO_3 (1.5 M aqueous solution), Boronic acid, DMF, 140 ºC, 5 mins; b) CDI, 1,4-dioxane, 140 ºC, 20min, 3-34%; vi)** For compounds **12** and **19-29; $Pd(PPh_3)_4$ (3 mol%), K_2CO_3 (1.5 M aqueous solution), Boronic acid, DMF, 140 ºC, 5 mins, 7-67% vii) For compound 16; 7, H_2, 5% Pd/C, MeOH, 15%**

3,5-dibromopyrazin-2-amine (5)

Pyrazin-2-amine (49.9 g, 52.5 mmol) was dissolved in MeCN (1 L), cooled to 5 °C then NBS (203 g, 1.1 mol) was added portion-wise over 2 hours. The temperature was allowed to increase to room temperature and the mixture stirred for 16 hours. The reaction mixture was concentrated then partitioned between EtOAc (500 mL) and water (500 mL), the mixture filtered through celite to remove the insoluble fraction. The organic fraction was retained and the aqueous layer was extracted with EtOAc (3 x 200 mL). The combined organics were washed with sodium hydroxide (1 M solution in water, 200 mL), water (200 mL) and brine (200 mL) then dried over $MgSO_4$ and concentrated *in vacuo*. The resultant crude residue was purified by column chromatography, eluting with 0-50% EtOAc/Pet. Ether 40-60 °C,

followed by re-crystallisation from EtOAc to give the title compound **5** (15.9 g, 12%) as yellow needles.

^{1}H NMR (500 MHz, DMSO-d_6): δ 8.15 (s, 1H), 7.00 (s, 2H).

LRMS (ES+): *m/z* (%) 251.9 [^{79}Br/ ^{79}Br M+H]$^+$,(60), 253.9 [^{79}Br/ ^{81}Br M+H]$^+$ (100), 255.9 [^{81}Br/ ^{81}Br M+H]$^+$ (50)

(R)-6-bromo-N2-(1-phenylethyl)pyrazine-2,3-diamine (6a)

3,5-dibromopyrazin-2-amine (4.0g, 15.8 mmol), (R)-1-phenylethanamine (2.24 mL, 17.4 mmol) and DIPEA (3.02 mL, 17.4 mmol) in ethanol (20 mL) was split into 2 batches, each of which were heated under microwave irradiation at 180 °C for 90 minutes. The reactions were cooled and combined then concentrated. The resulting slurry was partitioned between EtOAc (20 mL) and water (10 mL), the organic layer was washed with brine (200 mL) and concentrated *in* vacuo. The resultant crude residue was purified by column chromatography, eluting with 30-50% EtOAc/Pet. Ether 40-60 °C, to give the title compound **6a** (2.35g, 51%) as a clear oil.

^{1}H NMR (500 MHz, DMSO-d_6): δ 7.38-7.28 (m, 4H), 7.24 (m, 1H), 7.16 (s, 1H), 6.86 (d, *J* = 7.2 Hz, 1H),, 6.30 (s, 2H), 5.08 (m, 1H), 1.47 (d, *J* = 7.0 Hz, 3H)

LRMS (ES+): *m/z* (%) 293.0 [^{79}Br M+H]$^+$,(100), 295.0 [^{81}Br M+H]$^+$ (98)

6-bromo-N2-cyclohexylpyrazine-2,3-diamine (6b)

3,5-dibromopyrazin-2-amine (16.0 g, 63.2 mmol), DIPEA (19.2 mL, 110 mmol), cyclohexylamine (12.8 mL, 112 mmol) and *n*-butanol (120 mL) was split into 8 batches each of which were heated under microwave irradiation at 220 °C for 30 minutes. The reactions were cooled,combined and concentrated *in vacuo*. The resulting slurry was partitioned between EtOAc (200 mL) and water (200 mL), the organic layer was washed with water (200 mL) and brine (200 mL) and concentrated *in* vacuo. The resultant crude residue was purified by column chromatography, eluting with 0-50% EtOAc/Pet. Ether 40-60 °C to give the title compound **6b** (16.2 g, 95%) as a brown oil.

^{1}H NMR (500 MHz, DMSO--d_6): δ 7.13 (s, 1H), 6.25 (d, *J* = 7.15 Hz, 1H), 6.20 (s, 2H), 3.74-3.68 (m, 1H), 1.94-1.87 (m, 2H), 1.75-1.72 (m, 2H), 1.63-1.60 (m, 1H), 1.37-1.13 (m, 5H).

LRMS (ES+): *m/z* (%) 271.1 [^{79}Br M+H]$^+$,(100), 273.1 [^{81}Br M+H]$^+$ (98)

1-benzyl-6-(4-fluorophenyl)-1H-imidazo[4,5-b]pyrazin-2(3H)-one (9) (General method 1)

3,5-dibromopyrazin-2-amine (400mg, 1.58 mmol), benzylamine (189μL, 1.74 mmol) and DIPEA (302 μL, 1.74 mmol) in ethanol (2 mL) was heated under microwave irradiation at 180 °C for 90 minutes. The reaction was cooled to room temperature and concentrated *in vacuo*. The resulting slurry was partitioned between DCM (20 mL) and water (10 mL), the organic layer was washed with brine (10 mL) and concentrated *in* vacuo. The resultant crude residue was purified by column chromatography, eluting with 20% EtOAc/hexane to give **N2-benzyl-6-bromopyrazine-2,3-diamine (6f)** (281 mg, 64%) as a brown oil.

N2-benzyl-6-bromopyrazine-2,3-diamine (6f) (281 mg, 1.10 mmol), 4-fluorophenylboronic acid (154 mg, 1.10 mmol), tetrakis(triphenylphoshine)palladium (0) (58 mg, 0.05 mmol), potassium carbonate (414 mg, 3.0 mmol) in water (5 mL), and DMF (5 mL) were combined and heated under microwave irradiation at 160 °C for 5 minutes.The reaction was then cooled to room temperature and the mixture diluted with a 10 : 1 mixture of DCM : MeOH (10 mL).The mixture was poured onto an SCX-2 column and allowed to drip through then washed with 10 : 1 mixture of DCM : MeOH (10 mL). The SCX-2 column was then eluted with ammonia in MeOH (7 N, 20 mL) and the elutes concentrated *in vacuo* . The resultant brown solid was dissolved in 1,4-dioxane (2 mL) and CDI (140 mg, 0.86 mmol) added. The mixture was heated under microwave irradiation at 140 °C for 20 minutes, cooled to room temperature and concentrated *in vacuo*. The resultant crude residue was purified by column chromatography, eluting with 0-100% EtOAc /Pet. Ether 40-60 °C to give the title compound (**9**) (47 mg, 15%) was obtained as an off-white solid.

^{1}H NMR (500 MHz, DMSO--d_6): δ 12.19 (s, 1H), 8.53 (s, 1H), 8.09-8.06 (brs, 2H), 7.43-7.27 (m, 7H), 5.08 (s, 2H).

LRMS (ES+): *m/z* (%) 321.0 [M+H]$^+$ (100)

HRMS (m/z): [M+H]$^+$ calcd. for C18H14FN4O, 321.1146; found 321.1131.

(S)-6-(4-fluorophenyl)-1-(1-phenylethyl)-1H-imidazo[4,5-b]pyrazin-2(3H)-one (8)

Following the method outlined in General method 1, but using (S)-1-phenylethanamine (224 μL, 1.74 mmol) the title compound (**8**) (23 mg, 15%) was obtained as a yellow solid.

^{1}H NMR (500 MHz, DMSO--d_6): δ 12.16 (s, 1H), 8.51 (s, 1H), 8.06-8.03 (m, 2H), 7.50 (m, 2H), 7.37-7.26 (m, 5H), 5.74 (q, J = 7.3 Hz, 1H), 2.02 (d, J = 7.3 Hz, 3H).

1-(cyclopropylmethyl)-6-(4-fluorophenyl)-1H-imidazo[4,5-b]pyrazin-2(3H)-one (10)

Following the method outlined in General method 1, but using cyclopropylmethanamine (200μL, 1.74 mmol) , the title compound (**10**) (6 mg, 3%) was obtained as a yellow solid.

^{1}H NMR (500 MHz, DMSO--d_6): δ 12.09 (s, 1H), 8.51 (s, 1H), 8.09 (m, 2H), 7.33 (m, 2H), 3.76 (d, J = 7.1 Hz, 2H), 1.28 (m, 1H), 0.53 (m, 2H), 0.45 (m, 2H).

LRMS (ES+): *m/z* (%) 285.1 $[M+H]^+$ (100)

HRMS (m/z): $[M+H]^+$ calcd. for C15H14FN4O, 285.1146; found 285.1149

6-(4-fluorophenyl)-1-methyl-1H-imidazo[4,5-b]pyrazin-2(3H)-one (11)

Following the method outlined in General method 1, but using methylamine (870μL, 2M solution in MeOH, 1.74 mmol), the title compound (**11**) (6 mg, 3%) as an off-white solid.

^{1}H NMR (500 MHz, DMSO-d_6): δ 12.05 (s, 1H), 8.50 (s, 1H), 8.10 (dd, 2H, J = 5.5 and 9.0 Hz), 7.33 (t, 2H, J = 8.9 Hz), 5.08 (s, 2H).

HRMS (m/z): $[M+H]^+$ calcd. for C12H10FN4O, 245.0833; found 245.0826

(R)-6-phenyl-1-(1-phenylethyl)-1H-imidazo[4,5-b]pyrazin-2(3H)-one (14) (General method 2)

(R)-6-bromo-N2-(1-phenylethyl)pyrazine-2,3-diamine (**6a**) (100 mg, 0.35 mmol), phenyl boronic acid (44 mg, 0.35 mmol), tetrakis(triphenylphoshine)palladium (0) (20 mg, 0.018 mmol), potassium carbonate (700μL of a 1.5M solution in water, 1.05 mmol) and DMF (2 mL) were combined and heated under microwave irradiation at 160 °C for 5 minutes.The reaction was then cooled to room temperature and the mixture diluted with a 10 : 1 mixture of DCM : MeOH (10 mL).The mixture was poured onto an SCX-2 column and allowed to drip through then washed with 10 : 1 mixture of DCM : MeOH (10 mL). The SCX-2 column was then eluted with ammonia in MeOH (7 N, 20 mL) and the elutes concentrated *in vacuo* . The resultant brown solid was dissolved in 1,4-dioxane (2 mL) and CDI (140 mg, 0.86 mmol)

added. The mixture was heated under microwave irradiation at 140 °C for 20 minutes, cooled to room temperature and concentrated *in vacuo*. The resultant crude residue was purified by column chromatography, eluting with 0-100% EtOAc /Pet. Ether 40-60 °C to give the title compound (**14**) (21 mg, 18%) was obtained as a pale yellow solid.

^{1}H NMR (500 MHz, DMSO--d_6): δ 12.12 (s, 1H), 8.51 (s, 1H), 8.00 (d, *J* = 7.3 Hz, 2H), 7.55-7.47 (m, 4H), 7.42-7.34 (m, 3H), 7.27 (t, *J* = 7.3 Hz, 1H), 5.74 (q, *J* = 7.2 Hz, 1H), 2.03 (d, *J* = 7.3 Hz, 3H).

LRMS (ES+): *m/z* (%) 317.1 $[M+H]^+$ (97)

HRMS (m/z): $[M+H]^+$ calcd. for C19H17N4O, 317.1397; found 317.1394

(R)-1-(1-phenylethyl)-6-(4-(trifluoromethoxy)phenyl)-1H-imidazo[4,5-b]pyrazin-2(3H)-one (15)

Following the method outlined in General method 2, but using 4-(trifluoromethoxy)phenylboronic acid (82 mg, 0.4 mmol), the title compound (**15**) (57 mg, 34%) was obtained as a yellow solid.

^{1}H NMR (500 MHz, DMSO--d_6): δ 12.18 (s, 1H), 8.55 (s, 1H), 8.14-8.12 (m, 2H), 7.51-7.47 (m, 4H), 7.37-7.34 (m, 2H), 7.29-7.26 (m, 1H), 5.74 (q, J = 7.2 Hz, 1H), 2.02 (d, *J* = 7.3 Hz, 3H).

LRMS (ES+): *m/z* (%) 401.2 $[M+H]^+$ (100)

HRMS (m/z): $[M+H]^+$ calcd. for C20H16F3N4O2, 401.1220; found 401.1224

(R)-6-(3-fluorophenyl)-1-(1-phenylethyl)-1H-imidazo[4,5-b]pyrazin-2(3H)-one (17)

Following the method outlined in General method 2, but using 3-fluorophenylboronic acid (56 mg, 0.4 mmol), the title compound (**17**) (40 mg, 34%) was obtained as a yellow solid.

^{1}H NMR (500 MHz, DMSO--d_6): δ 12.18 (s, 1H), 8.58 (s, 1H), 7.87 (d, *J* = 7.8 Hz, 1H), 7.80 (d, *J* = 10.7 Hz, 1H), 7.55-7.49 (m, 3H), 7.37-7.34 (m, 2H), 7.29-7.21 (m, 2H), 5.75 (q, *J* = 7.2 Hz, 1H), 2.02 (d, *J* = 7.3 Hz, 3H).

LRMS (ES+): *m/z* (%) 335.1 $[M+H]^+$ (90)

HRMS (m/z): $[M+H]^+$ calcd. for C19H16FN4O, 335.1303; found 335.1299

(R)-N-(4-(2-oxo-3-(1-phenylethyl)-2,3-dihydro-1H-imidazo[4,5-b]pyrazin-5-yl)phenyl)methanesulfonamide (18)

Following the method outlined in General method 2, but using (R)-6-bromo-N2-(1-phenylethyl)pyrazine-2,3-diamine (100 mg, 0.35 mmol) and N-(3-4,4,5,5-tetramethyl-1,3,2-doxaborolan-2-yl)-phenylmethyanesulfonamide (81 mg, 0.27 mmol), the title compound (**18**) (7 mg, 3%) was obtained as a yellow solid.

^{1}H NMR (500 MHz, DMSO--d_6): δ 12.14 (s, 1H), 9.92 (s, 1H), 8.47 (s, 1H), 7.97 (s, 1H), 7.73 (d, *J* = 7.9 Hz, 1H), 7.56 (d, *J* = 7.7 Hz, 1H), 7.44 (t, *J* = 7.9 Hz, 1H), 7.37-7.34 (m, 2H), 7.29-7.24 (m, 2H), 5.74-5.70 (m, 1H), 3.05 (s, 3H), 2.05 (d, J = 7.3 *Hz*, 3H).

LRMS (ES+): *m/z* (%) 410.0 $[M+H]^+$ (100)

HRMS (m/z): $[M+H]^+$ calcd. for C20H20N5O3S, 410.1281; found 410.1272

(R)-6-bromo-1-(1-phenylethyl)-1H-imidazo[4,5-b]pyrazin-2(3H)-one 7a

(R)-6-bromo-N2-(1-phenylethyl)pyrazine-2,3-diamine (1g, 3.4 mmol), CDI (1.66 g, 10.2 mmol) and 1,4-dioxane (15 mL) were heated under microwave irradiation at 140 °C for 20 minutes and concentrated *in vacuo*. The resultant crude residue was purified by column chromatography,eluting with 1-5% MeOH/DCM, followed by trituration with EtOAc/hexane to give the title compound **7a** (947 mg, 87%) as a brown solid.

^{1}H NMR (500 MHz, DMSO--d_6): δ 12.31 (s, 1H), 8.05 (s, 1H), 7.38 (m, 2H), 7.35 (m, 2H), 7.28 (m, 1H), 5.62 (q, *J* = 7.3 Hz, 1H), 1.90 (d, *J* = 7.3 Hz, 3H)

LRMS (ES+): *m/z* (%) 319.0 $[^{79}Br\ M+H]^+$,(98), 321.0 $[^{81}Br\ M+H]^+$ (100)

HRMS (m/z): $[M+H]^+$ calcd. for C13H12^{79}BrN4O, 319.0189; found 319.0197

6-bromo-1-cyclohexyl-1H-imidazo[4,5-b]pyrazin-2(3H)-one, 7b

6-bromo-N2-cyclohexylpyrazine-2,3-diamine (16.25g, 63.2 mmol), CDI (12.3g, 75.9 mmol) and 1,4-dioxane (200 mL) were combined then heated to 80 °C under nitrogen atmosphere

for 16 h then cooled and concentrated *in vacuo*. The resultant crude residue was purified by column chromatography, eluting with 50% EtOAc/petrol ether 40-60 °C to give the title compound **7b** (5.8 g, 31%) as a white solid.

^{1}H NMR (500 MHz, DMSO--d_6): δ 12.2 (s, 1H), 8.02 (s, 1H), 4.17-4.11 (s, 1H), 2.18-2.11 (m, 2H), 1.85-1.76 (m, 4H), 1.68-1.66 (m, 1H), 1.41-1.33 (m, 2H) and 1.23-1.16 (m, 1H).

LRMS (ES+): *m/z* (%) 297.0 [^{79}Br M+H]$^+$,(98), 299.0 [^{81}Br M+H]$^+$ (100)

HRMS (m/z): [M+H]$^+$ calcd. for C11H14^{79}BrN4O, 297.0346; found 297.0360

1-cyclohexyl-6-(4-fluorophenyl)-1H-imidazo[4,5-b]pyrazin-2(3H)-one (12) (General method 3)

6-bromo-1-cyclohexyl-1H-imidazo[4,5-b]pyrazin-2(3H)-one **7b** (100 mg, 0.33 mmol), tetrakis(triphenylphoshine)palladium (0) (12 mg, 0.01 mmol), potassium carbonate (0.65 mL of a 1.5 M solution in water, 1.0 mmol), 4-fluorophenyl boronic acid (46 mg, 0.33 mmol), in DMF (2.0 mL) were combined and the mixture heated under microwave irradiation at 180 °C for 5 minutes. The reaction mixture was absorbed onto silica and purified by chromatography eluting with 50% EtOAc/Pet. Ether 40-60 °C to give the title compound (**12**) (17 mg, 16%)as a yellow solid.

^{1}H NMR (500 MHz, DMSO--d_6): δ 12.04 (s, 1H), 8.49 (s, 1H), 8.10-8.07 (m, 2H), 7.34 (t, J = 8.8 *Hz*, 2H), 4.28-4.22 (m, 1H), 2.37-2.29 (m, 2H), 1.88-1.80 (m, 4H), 1.71 (d, J = 12.8 *Hz*,1H), 1.44-1.36 (m, 2H), 1.29-1.22 (m, 1H).

LRMS (ES+): *m/z* (%) 313.2 [M+H]$^+$ (100)

HRMS (m/z): [M+H]$^+$ calcd. for C17H18FN4O, 313.1459; found 313.1458

1-cyclohexyl-6-phenyl-1H-imidazo[4,5-b]pyrazin-2(3H)-one (19)

Following the method outlined in General method 3, but using phenyl boronic acid (46 mg, 0.33 mmol), the title compound **(19)** (65 mg, 67%) was obtained as a pale yellow solid.

^{1}H NMR (500 MHz, DMSO-d_6): δ 12.04 (s, 1H), 8.50 (s, 1H), 8.03 (m, 2H), 7.50 (m, 2H), 7.40 (m, 1H), 4.25 (m, 1H), 2.33 (m, 2H), 1.86-1.80 (m, 4H), 1.72 (m, 1H), 1.42-1.36 (m, 2H), 1.26 (m, 1H).

LRMS (ES+): *m/z* (%) 295.1 $[M+H]^+$ (100)

HRMS (m/z): $[M+H]^+$ calcd. for C17H19N4O, 295.1553; found 295.1548

1-cyclohexyl-6-(4-methoxyphenyl)-1H-imidazo[4,5-b]pyrazin-2(3H)-one (20) Following the method outlined in General method 3, but using 4-methoxyphenyl boronic acid (36 mg, 0.33 mmol), the title compound **(20)** (36 mg, 34%) was obtained as a pale yellow solid.

^{1}H NMR (500 MHz, DMSO--d_6): δ 11.96 (s, 1H), 8.42 (s, 1H), 7.98 (d, *J* = 8.9 Hz, 2H), 7.06 d, *J* = 8.9 Hz, 2H), 4.25 (m, 1H), 3.81 (s, 3H), 2.33 (m, 2H), 1.86-1.80 (m, 4H), 1.72 (m, 1H), 1.42-1.36 (m, 2H), 1.26 (m, 1H).

LRMS (ES+): *m/z* (%) 325.2 (100)

HRMS (m/z): $[M+H]^+$ calcd. for C18H21N4O2, 325.1659; found 325.1643

1-cyclohexyl-6-(4-(hydroxymethyl)phenyl)-1H-imidazo[4,5-b]pyrazin-2(3H)-one (21)

Following the method outlined in General method 3, but using 4-hydroxymethylphenyl boronic acid (50 mg, 0.33 mmol), the title compound (**21**) (43 mg, 39%) was obtained as a yellow solid.

^{1}H NMR (500 MHz, DMSO-d_6): δ 12.01 (s, 1H), 8.47 (s, 1H), 7.98 (d, *J* = 8.2 Hz, 2H), 7.44 (d, *J* = 8.2 Hz, 2H), 5.27 (t, J = 5.7 *Hz*, 1H), 4.56 (d, J = 5.7 *Hz*, 2H), 4.28-4.23 (m, 1H), 2.37-2.30 (m, 2H), 1.88-1.80 (m, 4H), 1.71 (d, *J* = 12.1 Hz, 1H), 1.44-1.37 (m, 2H), 1.29-1.24 (m, 1H).

LRMS (ES+): *m/z* (%) 325.2 $[M+H]^+$ (99)

HRMS (m/z): $[M+H]^+$ calcd. for C18H21N4O2, 325.1659; found 325.1653

4-(3-cyclohexyl-2-oxo-2,3-dihydro-1H-imidazo[4,5-b]pyrazin-5-yl)-N-methylbenzamide (22)

Following the method outlined in General method 3, but using, 4-(N-methylbenzamide)boronic acid (59 mg, 0.33 mmol), the title compound (**22**) (21 mg, 18%) was obtained as a yellow solid.

^{1}H NMR (500 MHz, DMSO--d_6): δ 12.10 (s, 1H), 8.69 (s, 1H), 8.58 (s, 1H), 8.53-8.51 (m, 1H), 8.12 (d, *J* = 8.4 Hz, 2H), 7.95 (d, *J* = 8.4 Hz, 2H), 2.82 (d, *J* = 4.5 Hz, 3H), 2.37-2.24 (m, 1H), 1.89-1.81 (m, 4H), 1.72 (d, *J* = 12.3 Hz, 1H), 1.45-1.37 (m, 2H), 1.30-1.25 (m, 1H).

LRMS (ES+): *m/z* (%) 352.2 $[M+H]^+$ (99)

HRMS (m/z): $[M+H]^+$ calcd. for C19H22N5O2, 352.1768; found 352.1781

1-cyclohexyl-6-(4-((dimethylamino)methyl)phenyl)-1H-imidazo[4,5-b]pyrazin-2(3H)-one (23)

Following the method outlined in General method 3, but using 4-[(dimthylamino),methyl]boronic acid hydrochloride (67 mg, 0.33 mmol), the title compound (**23**) (13 mg, 11%) was obtained as a pale yellow solid.

^{1}H NMR (500 MHz, DMSO--d_6): δ 12.02 (s, 1H), 8.47 (s, 1H), 7.97 (d, *J* = 8.2 Hz, 2H), 7.41 (d, *J* = 8.2 Hz, 2H), 4.26-4.25 (m, 1H), 2.37-2.30 (m, 2H), 2.17 (s, 6H), 1.88-1.80 (m, 4H), 1.71 (d, *J* = 12.9 Hz, 1H), 1.42-1.36 (m, 2H), 1.27-1.24 (m, 1H).

LRMS (ES+): *m/z* (%) 352.0 $[M+H]^+$ (98)

HRMS (m/z): $[M+H]^+$ calcd. for C20H26N5O, 352.2132; found 352.2139

1-cyclohexyl-6-(3-(hydroxymethyl)phenyl)-1H-imidazo[4,5-b]pyrazin-2(3H)-one (24)

Following the method outlined in General method 3, but using 3-(hydroxymethyl)phenyl boronic acid (49 mg, 0.33 mmol), the title compound (**24**) (19 mg, 17%) was obtained as a yellow solid.

^{1}H NMR (500 MHz, DMSO--d_6): δ 11.84 (s, 1H), 8.27 (s, 1H), 7.70 (d, *J* = 7.2 Hz, 1H), 7.44 (s, 1H), 7.27 (t, *J* = 6.7 Hz, 1H), 7.17 (d, J = 7.0 *Hz*, 1H), 5.10 (s, 1H), 4.39 (d, J = 4.3 *Hz*, 1H), 4.07 (brs, 1H), 2.16-2.11 (m, 2H), 1.69-1.61 (m, 4H), 1.52 (d, *J* = 11.2 Hz, 1H), 1.23-1.20 (m, 2H), 1.07-1.05 (m, 1H).

LRMS (ES+): *m/z* (%) 325.0 $[M+H]^+$ (100)

HRMS (m/z): $[M+H]^+$ calcd. for C18H21N4O2, 325.1659; found 325.1668

N-(3-(3-cyclohexyl-2-oxo-2,3-dihydro-1H-imidazo[4,5-b]pyrazin-5-yl)phenyl)acetamide (25)

Following the method outlined in General method 3, but using 3-phenylacetamide boronic acid (58 mg, 0.33 mmol), the title compound (**25**) (29 mg, 24%) was obtained as a yellow solid.

^{1}H NMR (500 MHz, DMSO--d_6): δ 12.05 (s, 1H), 10.08 (s, 1H), 8.38 (s, 1H), 8.24 (s, 1H), 7.67-7.64 (m, 2H), 7.41 (t, *J* = 7.9 Hz, 1H), 4.28-4.22 (m, 1H), 2.39-2.32 (m, 2H), 1.89-1.80 (m, 4H), 1.71 (d, *J* = 12.2 Hz, 1H), 1.44-1.29 (m, 3H).

LRMS (ES+): *m/z* (%) 352.2 $[M+H]^+$ (100)

HRMS (m/z): $[M+H]^+$ calcd. for C19H22FN5O2, 352.1768; found 352.1774

1-cyclohexyl-6-(2-fluorophenyl)-1H-imidazo[4,5-b]pyrazin-2(3H)-one (26)

Following the method outlined in General method 3, but using 2-fluoro boronic acid (40 mg, 0.33 mmol), the title compound **(26)** (46 mg, 44%) was obtained as a pale yellow solid.

^{1}H NMR (500 MHz, DMSO--d_6): δ 12.12 (s, 1H), 8.30 (s, 1H), 7.93-7.90 (m, 1H), 7.47 (brs, 1H), 7.40-7.34 (m, 2H), 4.11 (d, *J* = 4.5 Hz, 1H), 2.37-2.27 (m, 2H), 1.86-1.80 (m, 4H), 1.69 (d, *J* = 12.3 Hz, 1H), 1.40-1.38 (m, 2H), 1.25-1.21 (m, 1H).

LRMS (ES+): *m/z* (%) 313.1 $[M+H]^+$ (100)

HRMS (m/z): $[M+H]^+$ calcd. for C17H18FN4O, 313.1459; found 313.1461

1-cyclohexyl-6-(2-methoxyphenyl)-1H-imidazo[4,5-b]pyrazin-2(3H)-one (27)

Following the method outlined in General method 3, but using 2-methoxyphenyl boronic acid (56 mg, 0.33 mmol), the title compound **(27)** (22 mg, 19%) was obtained as a yellow solid.

^{1}H NMR (500 MHz, DMSO--d_6): δ 12.08 (s, 1H), 8.09 (s, 1H), 7.59-7.41 (m, 4H), 4.26-4.20 (m, 1H), 3.35 (s, 3H), 2.30-2.23 (m, 2H), 1.85-1.80 (m, 4H), 1.68 (d, *J* = 12.3 Hz, 1H), 1.42-1.34 (m, 2H), 1.23-1.15 (m, 1H).

1-cyclohexyl-6-(2-(hydroxymethyl)phenyl)-1H-imidazo[4,5-b]pyrazin-2(3H)-one (28)

Following the method outlined in General method 3, but using 2-methoxyphenyl boronic acid (55 mg, 0.33 mmol), the title compound (**28**) (58 mg, 53%) was obtained as a pale yellow solid.

^{1}H NMR ((500 MHz, DMSO--d_6): δ 12.06 (s, 1H), 8.09 (s, 1H), 7.66 (d, *J* = 7.0 Hz, 1H), 7.46-7.44 (m, 2H), 7.39-7.36 (m, 1H), 5.16 (s, 1H), 4.65 (d, *J* = 4.0 Hz, 2H), 4.23-4.19 (m, 1H), 2.29-2.22 (m, 2H), 1.84-1.78 (m, 4H), 1.67 (d, *J* = 11.2 Hz, 1H), 1.38-1.35 (m, 2H), 1.22-1.17 (m, 1H).

LRMS (ES+): *m/z* (%) 325.0 $[M+H]^+$ (100)

HRMS (m/z): $[M+H]^+$ calcd. for C18H21N4O2, 325.1659; found 325.1651

1-cyclohexyl-6-(pyridin-4-yl)-1H-imidazo[4,5-b]pyrazin-2(3H)-one (29) Following the method outlined in General method 3, but using 4-pyridylboronic acid (41 mg, 0.33 mmol), the title compound (**29**) (7 mg, 7%) was obtained as a yellow solid.

^{1}H NMR (500 MHz, DMSO--d_6): δ 12.22 (s, 1H), 8.69-8.68 (m, 3H), 8.04-8.03 (m, 2H), 4.30-4.23 (m, 1H), 2.37-2.29 (m, 2H), 1.89-1.81 (m, 4H), 1.72 (d, *J* = 12.4 Hz, 1H), 1.45-1.37 (m, 2H), 1.32-1.24 (m, 1H).

1-cyclohexyl-6-(pyridin-3-yl)-1H-imidazo[4,5-b]pyrazin-2(3H)-one (30) Following the method outlined in General method 3, but using 3-pyridyl boronic acid (41 mg, 0.33 mmol), the title compound (**30**) (13 mg, 13%) was obtained as a yellow solid.

^{1}H NMR (500 MHz, DMSO--d_6): δ 12.13 (s, 1H), 9.23 (s, 1H), 8.61-8.58 (m, 2H), 8.39 (d, *J* = 7.5 Hz, 1H), 7.53 (s, 1H), 4.28-4.24 (m, 1H), 2.37-2.29 (m, 2H), 1.88-1.81 (m, 4H), 1.71 (d, *J* = 12.1 Hz, 1H), 1.42-1.37 (m, 2H), 1.30-1.25 (m, 1H).

LRMS (ES+): *m/z* (%) 296.2 $[M+H]^+$ (100)

(R)-1-(1-phenylethyl)-1H-imidazo[4,5-b]pyrazin-2(3H)-one (16) To a mixture of (R)-6-bromo-1-(1-phenylethyl)-1H-imidazo[4,5-b]pyrazin-2(3H)-one (100 mg, 0.31 mmol) in MeOH/DCM (8:2, 10.0 mL) at room temperature was added 5% Pd/C (30.0 mg),the mixture

degassed and placed under an atmosphere of nitrogen gas. The mixture was degassed, placed under an atmosphere of hydrogen gas, and stirred vigorously for 17 hours. The reaction was then filtered through a celite pad, the pad washed with DCM (20 mL), MeOH (20 mL) and filtrate concentrated *in vacuo*. The crude solid was triturated with 1:1 hexane/diethyl ether, filtered and dried *in vacuo* to give the title compound (**16**) (11 mg, 15%) as an off-white solid.

^{1}H NMR (300 MHz, DMSO-d_6): 7.65 (brs, 1H), 7.53 (brs, 1H), 7.38 (d, 2H, J = 4.5 Hz), 7.30 (t, 2H, 4.4 Hz), 7.22 (t, 2H, 4.4 Hz), 5.62 (q, 1H, 4.4 Hz), 4.13 (brs, 1H), 1.90 (d, 2H, J = 4.4 Hz).

HRMS (m/z): $[M+H]^+$ calcd. for C13H13N4O, 241.1084; found 241.1092.

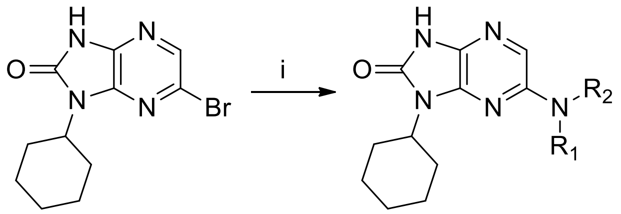

7a   31-33

Scheme 2: i. Pd_2dba_3 (5 mol%), amine (2 eq.), Rac-Binap (10 mol%), KO^{t}Bu, 1,4-dioxane, 160 °C, µW , 20 mins., 5-24%

1-cyclohexyl-6-(piperidin-1-yl)-1H-imidazo[4,5-b]pyrazin-2(3H)-one (31) (General method 4)

6-bromo-1-cyclohexyl-1H-imidazo[4,5-b]pyrazin-2(3H)-one (100 mg, 0.33 mmol), tris(dibenzylideneacetone)dipalladium (16 mg, 0.017 mmol), potassium *tert*-butoxide (114 mg, 1.0 mmol), (+/-)-2,2'-bis(diphenylphosphino)-1,1'-binaphthyl (22 mg, 0.034 mmol), piperidine (67 µl, 0.68 mmol) and 1,4-dioxane (2.0 mL) were combined, heated under microwave irradiation at 180 °C for 5 minutes.The reaction was cooled to room temperature, absorbed onto silica and purified by column chromatography, eluting with 50% EtOAc /hexane to give the title compound (**31**) (25 mg, 24%) as a yellow solid.

^{1}H NMR (500 MHz, DMSO--d_6): δ 11.36 (s, 1H), 7.45 (s, 1H), 4.16-4.09 (m, 1H), 3.38-3.32 (m, 2H), 2.52-2.50 (m, 2H), 2.27-2.19 (m, 2H), 1.83 (d, J = 13.0 Hz, 2H), 1.73-1.67 (m, 3H), 1.59 (brs, 6H), 1.39-1.31 (m, 2H), 1.21-1.13 (m, 1H).

LRMS (ES+): *m/z* (%) 302.2 $[M+H]^+$ (100)

HRMS (m/z): $[M+H]^+$ calcd. for C16H24N5O, 302.1975; found 302.1973

1-cyclohexyl-6-morpholino-1H-imidazo[4,5-b]pyrazin-2(3H)-one (32)

Following the method outlined in General method 4, but using morpholine (59 mg, 0.68 mmol), the title compound (**32**) (13 mg, 13%) was obtained as a pale yellow solid.

^{1}H NMR (500 MHz, DMSO--d_6): δ 11.46 (s, 1H), 7.46 (s, 1H), 4.16-4.11 (m, 1H), 3.76-3.74 (m, 4H), 3.34-3.32 (m, 4H), 2.26-2.19 (m, 2H), 1.83 (d, J = 13.0 Hz, 2H), 1.73-1.66 (m, 3H), 1.39-1.31 (m, 2H), 1.23-1.17 (m, 1H).

LRMS (ES+): *m/z* (%) 304.2 $[M+H]^+$ (100)

HRMS (m/z): $[M+H]^+$ calcd. for C15H22N5O2, 304.1768; found 304.1771

1-cyclohexyl-6-(4-methylpiperazin-1-yl)-1H-imidazo[4,5-b]pyrazin-2(3H)-one (33)

Following the method outlined in General method 4, but using N-methylpiperazine (75 µl, 0.68 mmol), the title compound (**33**) (5 mg, 5%) was obtained as a grey solid.

^{1}H NMR (500 MHz, DMSO--d_6): δ 11.42 (s, 1H), 7.46 (s, 1H), 4.13 (brs, 1H), 2.44 (s, 2H), 2.22 (s, 3H), 1.83 (d, J = 12.2 Hz, 2H), 1.73-1.66 (m, 3H), 1.39-1.31 (m, 2H), 1.22-1.17 (m, 1H). (missing 2 protons due to overlap with solvent)

LRMS (ES+): *m/z* (%) 317.1 $[M+H]^+$ (100)

HRMS (m/z): $[M+H]^+$ calcd. for C16H25N6O, 317.2084; found 317.2089

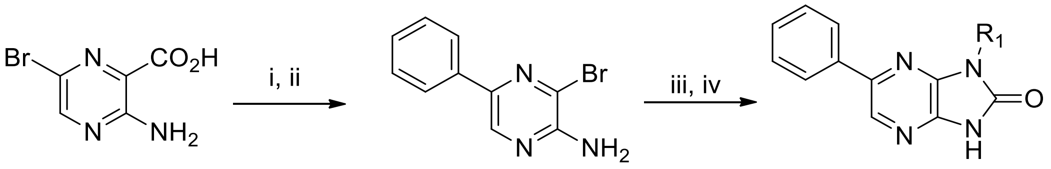

34 35 36-42

Scheme 3 i) phenylboronic acid, $Pd(PPh_3)_4$, K_2CO_3 (2M sol^n), DMF, μW, 140 °C, 10 min, 100%; ii) NaOAc, AcOH, Br_2, rt, 16 h, 63%; iii) Amine, DIPEA, n-BuOH, μW, 220°C, 1h; iv) CDI, dioxane, μW, 130 °C, 10 min, 2-71% for 2 steps.

3-Bromo-5-phenylpyrazine-2-amine (35)

3-Amino-6-bromopyrazine-2-carboxylic acid **34** (1.7 g, 8 mmol), DMF (6 mL), potassium carbonate (6 mL of a 2M solution in water, 12 mmol), tetrakis(triphenylphoshine)palladium (0) (10 mg, 0.008 mmol) and phenyl boronic acid were added sequentially to a 20 mL process vial and heated under microwave irradiation at 140 °C for 10 minutes. Two reactions were performed on this scale and combined to be worked up (16 mmol total reaction scale). The reaction mixture was partitioned between EtOAc and water, the organic layer washed (water, 2 x 20 mL) and the aqueous layer acidified to pH 4 with hydrochloric acid (10% v/v). A yellow precipitate was obtained which was re-dissolved in DCM and washed (water, 2 x 20 mL), the organic layer was dried over $MgSO_4$, filtered and the solvent removed *in vacuo* to give 3-Amino-6-phenylpyrazine-2-carboxylic acid (4 g, 100%) as a pale yellow solid.

1H NMR (500 MHz, MeOD) δ 8.78 (s, 1H), 8.04-8.02 (m, 2H), 7.48 (t, J = 7.9 Hz, 2H), 7.40 (tt, J = 7.1 and 1.2 Hz, 1H).

LRMS (ES+): *m/z* (%)216.0 $[M+H]^+$ (100)

3-Amino-6-phenylpyrazine-2-carboxylic acid (4 g, 16 mmol) and sodium acetate (2.7 g, 33 mmol) were stirred in acetic acid (30 mL) at rt for 30 min, bromine (0.93 mL, 18 mmol) in acetic acid (10 mL) was added drop wise over a period of 30 mins and the reaction stirred for 16 h. The reaction was quenched with water to give a tan precipitate which was collected and dried in a freeze drier to give the title compound **35** (2.52 g, 63%) as a tan solid.

^{1}H NMR (500 MHz, DMSO--d_6): δ 8.62 (s, 1H), 7.96 (d, J = 7.3 Hz, 2H), 7.46 (t, J = 7.3 Hz, 1H), 7.37 (t, J = 7.3 Hz, 2H), 6.90 (brs, 2H).

LRMS (ES+): *m/z* (%) 250.0 $[^{79}Br\ M+H]^+$,(100), 252.0 $[^{81}Br\ M+H]^+$ (98)

1-Isopropyl-6-phenyl-1*H*-imidazo[4,5-*b*]pyrazin-2(3H)-one (36) (General method 5)

3-Bromo-5-phenylpyrazine-2-amine (0.5 mmol), isopropylamine (61 µL, 44 mg, 0.75 mmol), DIPEA (0.1 mL) and ^{n}BuOH (0.5 mL) were heated under microwave irradiation at 220 °C for 1 hour. The reaction was diluted with EtOAc (10 mL), washed with H_20 (3 x 10 mL), the organic layer dried over $MgSO_4$, filtered and the solvent removed *in vacuo*. Column chromatography eluting with DCM to DCM:MeOH (95:5) afforded the desired substituted 6-phenylpyrazine-2,3-diamine that was reacted on without further purification. Substituted 6-phenylpyrazine-2,3-diamine (0.5 mmol), CDI (0.6 mmol) and 1,4-dioxane (1 mL) were heated under microwave irradiation at 130 °C for 10 minutes. The crude reaction mixture was partitioned between EtOAc and water, the organic layer dried over $MgSO_4$, filtered and the solvent removed *in vacuo*. The resultant crude residue was purified by column chromatography, eluting with EtOAc to give the title compound (**36**) (13 mg, 10%) as a colourless solid.

1H NMR (500 MHz, DMSO--d_6): δ 12.00 (brs, 1H), 8.50 (s, 1H), 8.04 (d, *J* = 7.3 Hz, 2H), 7.51 (t, *J* = 7.3 Hz, 2H), 7.44-7.39(m, 1H), 4.69 (sep, *J* = 6.8 Hz, 1H), 1.57 (d, *J* = 6.8 Hz, 6H).

LRMS (ES+): *m/z* (%) 255.1 $[M+H]^+$ (100)

1,6-Diphenyl-1*H*-imidazo[4,5-*b*]pyrazin-2(3*H*)-one (37)

Following the method outlined in General method 5, but using aniline (68 µL, 70 mg, 0.75 mmol), the title compound (**37**) (3 mg, 2%) was obtained as a pale yellow solid.

1H NMR (500 MHz, $CDCl_3$): δ 8.26 (brs, 1H), 7.69 (s, 1H), 7.47 (brs, 4H,), 7.24 (m, 1H), 6.89 (brs, 5H)

LRMS (ES+): *m/z* (%) 290.1 $[M+H]^+$ (100)

6-Phenyl-1-(tetrahydro-2*H*-pyran-4-yl)-1*H*-imidazo[4,5-*b*]pyrazin-2(3*H*)-one (38)

Following the method outlined in General method 5, but using 4-aminotetrahydropyran (81 µL, 79 mg, 0.75 mmol), the title compound (**38**) (65 mg, 44%) was obtained as a beige solid.

^{1}H NMR (500 MHz, $CDCl_3$): δ 8.43 (s, 1H), 8.39 (brs, 1H), 8.01 (m, 2H,), 7.53 (t, *J* = 7.3 Hz, 2H), 7.46 (tt, *J* = 7.3 and 1.1 Hz, 1H), 4.73-4.65 (m, 1H), 4.20 (dd, *J* = 11.0 and 4.5 Hz, 2H), 3.60 (t, *J* = 11.0 and 10.6 Hz, 2H), 2.90 (ddd, *J* = 25.0, 12.4 and 4.5 Hz, 2H), 1.84 (dd, *J* = 12.4 and 2.0 Hz, 2H)

LRMS (ES+): *m/z* (%) 297.2 $[M+H]^+$ (100)

1-(1-Methoxypropan-2-yl)-6-phenyl-1*H*-imidazo[4,5-*b*]pyrazin-2(3*H*)-one (39)

Following the method outlined in General method 5, but using 1-methoxybutan-2-amine (77 mg, 0.75 mmol), the title compound (**39**) (85 mg, 60%) was obtained as a colourless solid

^{1}H NMR (500 MHz, $CDCl_3$): δ 8.90 (brs, 1H), 8.41 (s, 1H), 7.99-7.97-(m, 2H), 7.51 (t, *J* = 7.3 Hz, 2H), 7.44 (t, *J* = 7.3 Hz, 1H), 4.98-4.94 (m, 1H), 4.27 (t, *J* = 10.1 Hz, 1H), 3.68 (dd, *J* = 10.1 and 5.2 Hz, 1H), 3.37 (s, 3H), 1.65 (d, *J* = 7.1 Hz, 3H).

LRMS (ES+): *m/z* (%) 285.1 $[M+H]^+$ (100)

HRMS (m/z): [MH+] calcd. For $C_{15}H_{16}N_4O_2$, 285.1346; found, 285.1336.

1-(1-Hydroxybutan-2-yl)-6-phenyl-1*H*-imidazo[4,5-*b*]pyrazin-2(3*H*)-one (40)

Following the method outlined in General method 5, but using 4-aminobutanol (71 µL, 67 mg, 0.75 mmol), the title compound (**40**) (100 mg, 71%) was obtained as a colourless solid;

^{1}H NMR (500 MHz, $CDCl_3$): δ 7.87 (s, 1H), 7.82-7.77 (m, 2H), 7.40 (t, *J* = 7.3 Hz, 2H), 7.30 (t, *J* = 7.3 Hz, 1H), 4.14-4.09 (m, 1H), 3.86 (dd, *J* = 11.4 and 3.3 Hz, 1H), 3.67 (dd, *J* = 11.4 and 6.9 Hz, 1H), 1.77-1.62 (m, 2H), 1.03 (t, *J* = 7.5 Hz, 3H),

LRMS (ES+): *m/z* (%) 285.2 $[M+H]^+$ (100)

HRMS (m/z): $[MH^+]$ calcd. For $C_{15}H_{16}N_4O_2$, 285.1346; found, 285.1334.

1-(2-(Dimethylamino)ethyl)-6-phenyl-1*H*-imidazo[4,5-*b*]pyrazin-2(3*H*)-one (41)

Following the method outlined in General method 5, but using N^1,N^1-dimethylethane-1,2-diamine (66 mg, 0.75 mmol), the title compound (**41**) (10 mg, 8%) was obtained as a colourless solid;

^{1}H NMR (500 MHz, $CDCl_3$): δ 8.00 (s, 1H), 7.90-7.89 (m, 2H), 7.49 (t, *J* = 7.3 Hz, 2H), 7.44 (tt, *J* = 7.3 Hz, 1H), 4.21 (t, *J* = 5.9 Hz, 2H), 3.01 (t, *J* = 5.9 Hz, 2H, CH_2), 2.49 (s, 6H, 2 x CH_3).

LRMS (ES+): *m/z* (%) 284.1 $[M+H]^+$

HRMS (m/z): [MH+] calcd. For $C_{15}H_{17}N_5O$, 284.1506; found, 284.1492.

1-(1-Methylpiperidin-4-yl)-6-phenyl-1*H*-imidazo[4,5-*b*]pyrazin-2(3*H*)-one (42)

Following the method outlined in General method 5, but using 1-methylpiperidin-4-amine (85 mg, 0.75 mmol), the title compound (**42**) (6.7 mg, 4%) was obtained as a beige solid.

^{1}H NMR (500 MHz, MeOD): δ 8.43 (s, 1H), 8.08 (d, *J* = 7.4 Hz, 2H), 7.49 (t, *J* = 7.4 Hz, 2H), 7.41 (tt, *J* = 7.4 and 1.2 Hz, 1H), 4.44 (tt, *J* = 12.1 and 4.0 Hz, 1H), 3.11 (d, *J* = 12.1, 2H), 2.92 (dd, *J* = 12.7 and 3.7 Hz, 1H), 2.88 (dd, *J* = 12.7 and 3.7 Hz, 1H), 2.39 (s, 3H), 2.29 (td, *J* = 12.1 and 2.3 Hz, 2H), 1.90 (d, *J* = 12.1 Hz, 2H),

LRMS (ES+): *m/z* (%) 310.1 $[M+H]^+$ (100)

HRMS (m/z): [MH+] calcd. For $C_{17}H_{19}N_5O$, 310.1662; found, 310.1671.

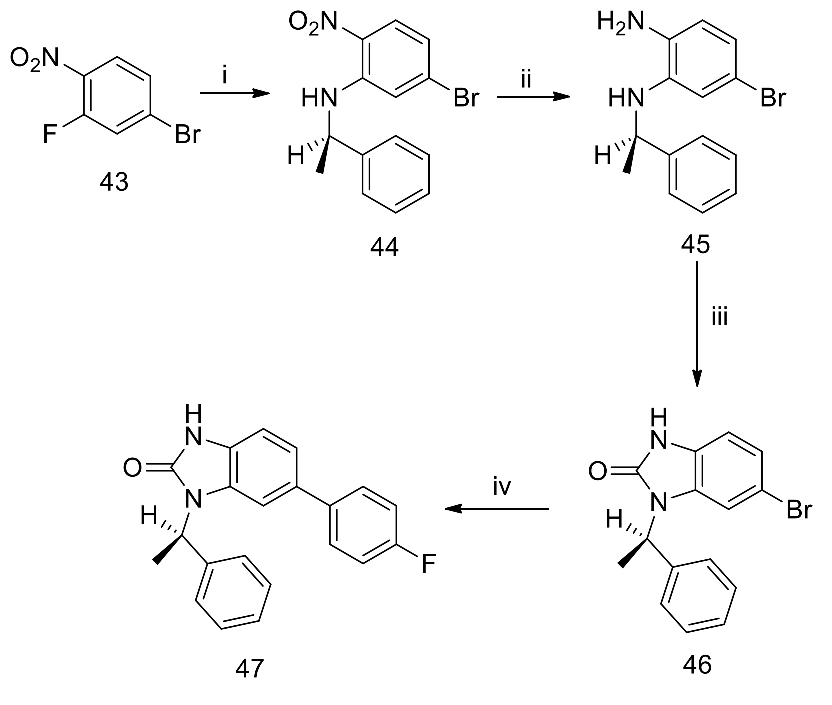

Scheme 4: i. (R)-Methylbenzylamine, Cs_2CO_3, DMF, 120 °C; ii. Charcoal 200% w/w, $NaBH_4$, THF, Water, rt., 16 h, 34% for 2 steps; iii. CDI, 1,4-dioxane, 140 °C, 20 mins, 61%; iv. $Pd(PPh_3)_4$ (5 mol%), K_2CO_3 (1.5 M aqueous solution), boronic acid, DMF, 140 °C, 5 mins., 44%.

(R)-5-bromo-N1-(1-phenylethyl)benzene-1,2-diamine (45)

4-bromo-2-fluoro-1-nitrobenzene (1g, 4.5 mmol), (R)-1-phenylethanamine (580μL, 4.5 mmol) and cesium carbonate (1.47g, 4.5 mmol) in DMF (4 mL) were heated under microwave irradiation at 120°C for 5 minutes. The solvent was removed *in vacuo* to give a crude residue which was taken up in EtOAc, washed (water), dried ($MgSO_4$) and concentrated *in vacuo* to give (R)-5-bromo-2-nitro-N-(1-phenylethyl)aniline **(44)** as an orange oil. The orange oil was taken up in THF (20 mL) and water (10 mL), charcoal (3g) added and the mixture stirred for 10 minutes. Sodium borohydride (1.5g, 39.7 mmol) was added portionwise and the reaction stirred for 66 hours. The reaction mixture was filtered through a pad of celite and the filtrate concentrated *in vacuo* to give a crude residue which was purified by column chromatography, eluting with 0-100% diethyl ether/hexane, to give the title compound **45** (446 mg, 34%) as a yellow oil.

1H NMR (500 MHz, DMSO--d_6): δ 7.36 (m, 2H), 7.31 (m, 2H), 7.20 (m, 1H), 6.43 (m, 2H), 6.20 (s, 1H), 5.16 (d, *J* = 6.5 Hz); 4.88 (s, 2H), 4.46 (m, 1H), 1.44 (d, *J* = 6.6 Hz, 3H)

(R)-6-bromo-1-(1-phenylethyl)-1H-benzo[d]imidazol-2(3H)-one (46)

(R)-5-bromo-N1-(1-phenylethyl)benzene-1,2-diamine **(45)** (446 mg, 1.53 mmol), and CDI (300 mg, 1.85 mmol) in 1,4-dioxane (5 mL) was heated under microwave irradiation at 140°C for 20 minutes. The solvent was removed *in vacuo* to give a crude residue which was purified by column chromatography, eluting with 0-100% diethyl ether/Pet. Ether 40-60°C to give the title compound **46** (297 mg, 61%) as a pale orange solid.

1H NMR (500 MHz, DMSO--d_6): 11.16 (s, 1H), 7.36 (m, 4H), 7.30 (m, 1H), 7.10 (dd, *J* = 1.7 and 8.3 Hz, 1H), 6.99 (d, *J* = 1.8 Hz, 1H), 6.94 (d, *J* = 8.3 Hz, 1H), 5.66 (q, *J* = 7.1 Hz, 1H), 1.81 (d, *J* = 7.3 Hz, 6H).

LRMS (ES+): *m/z* (%) 317.0 [^{79}Br M+H]$^+$,(98), 319.0 [^{81}Br M+H]$^+$ (100)

HRMS (m/z): [M+H]$^+$ calcd. for C15H14^{79}BrN2O, 317.0284; found 317.0286

(R)-6-(4-fluorophenyl)-1-(1-phenylethyl)-1H-benzo[d]imidazol-2(3H)-one (47)

(R)-6-bromo-1-(1-phenylethyl)-1H-benzo[d]imidazol-2(3H)-one **(46)** (95 mg, 0.3 mmol), phenyl boronic acid (46 mg, 0.33 mmol), tetrakis(triphenylphoshine)palladium (0) (17 mg, 0.015 mmol), potassium carbonate (0.6 mL mL of a 1.5 M aqueous solution, 0.9 mmol), and DMF (2 mL mL) were combined and heated under microwave irradiation at 140 °C for 5 mins. The reaction mixture was concentrated *in vacuo* to give a crude residue which was purified by column chromatography, eluting with 0-100% diethyl ether/petroleum ether 40-60°C to give the title compound **47** (44 mg, 44%) as a pale orange solid.

1H NMR (500 MHz, DMSO--d_6): 11.01 (s, 1H), 7.51 (m, 2H), 7.41 (m, 2H), 7.36 (m, 2H), 7.27 (m, 1H), 7.21 (m, 3H), 7.05 (d, *J* = 8.1 Hz, 1H), 7.03 (d, *J* = 1.5 Hz, 1H), 5.73 (q, *J* = 7.3 Hz, 1H), 1.88 (d, *J* = 7.3 Hz, 6H).

LRMS (ES+): *m/z* (%) 333.2 $[M+H]^+$ (100)

HRMS (m/z): $[M+H]^+$ calcd. for C21H18FN2O, 333.1398; found 333.1410

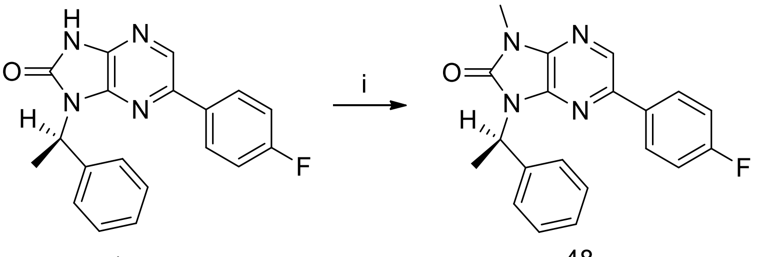

1 48

Scheme 5: i. NaH (60% w/w in mineral oil, 1.1 eq.), MeI (1.5 eq.), DMF, rt. 3 h, 38%.

(R)-5-(4-fluorophenyl)-1-methyl-3-(1-phenylethyl)-1H-imidazo[4,5-b]pyrazin-2(3H)-one (48)

To a stirred suspension of 60% sodium hydride (7 mg, 0.17 mmol) in DMF (1 mL) under an inert atmosphere, was added (R)-6-(4-fluorophenyl)-1-(1-phenylethyl)-1H-imidazo[4,5-b]pyrazin-2(3H)-one (**1**) (50 mg, 0.15 mmol) in DMF (1 mL) and the reaction was stirred for 20 minutes. Iodomethane (14μL, 0.23 mmol) was added to the reaction mixture and stirring continued for 4 hours. The reaction mixture was then concentrated *in vacuo* to give a crude

residue which was purified by column chromatography, eluting with 0-100% diethyl ether/Pet. Ether 40-60°C to give the title compound **48** (20 mg, 38%) as an off-white solid.

1H NMR (500 MHz, DMSO--*d*$_6$): 8.58 (s, 1H), 8.06 (m, 2H), 7.51 (d, J = 7.7 Hz, 2H), 7.36-7.24 (m, 5H), 5.79 (q, *J* = 7.3 Hz, 1H), 2.02 (d, *J* = 7.3 Hz, 6H).

LRMS (ES+): *m/z* (%) 349.2 $[M+H]^+$ (100)

HRMS (m/z): $[M+H]^+$ calcd. for C20H18FN4O, 349.1459; found 349.1455

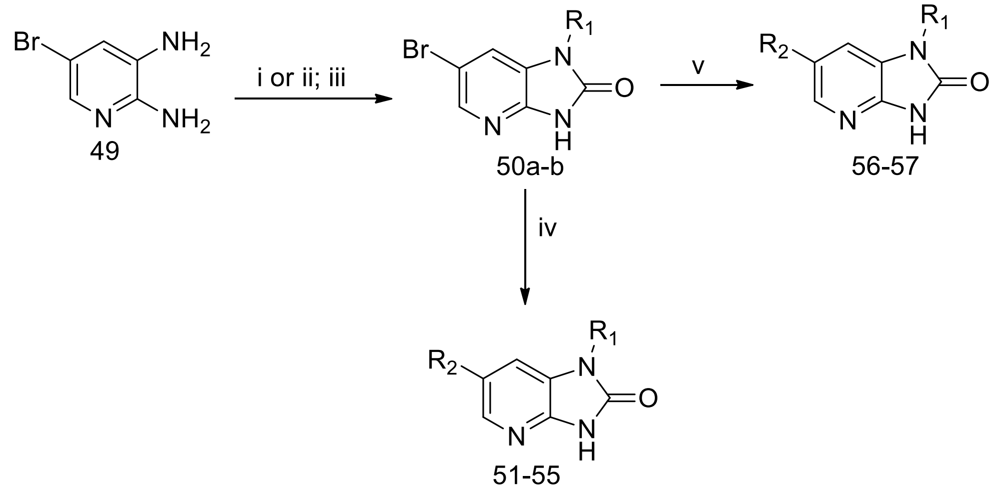

Scheme 6: i) a) Cyclohexanone, AcOH, DCM; b) $Na(OAc)_3BH$, 63%; c) CDI, MeCN, 75% ii) a) $PhC(CH_3)_2Cl$, , Et_3N, THF; 9% b) CDI, MeCN, 69%; iii) $ArB(OR)_2$, K_3PO_4, $Pd(PPh_3)_4$, DMF:H_2O (3:1), 130°C, mw, 3-69%; v) S-PHOS, NaOtBu, $(R^2)_2NH$, Pd_2dba_3, toluene, 120°C, mw, 37-43%

6-bromo-1-cyclohexyl-1H-imidazo[4,5-b]pyridin-2(3H)-one 50a

To a stirred suspension of 2,3-amino-5-bromopyridine (2.82g, 15 mmol) in DCM (30 mL) was added cyclohexanone (1.55 mL, 15 mmol) and acetic acid (0.86 mL, 15 mmol) and the reaction stirred for 1 hour. To the reaction mixture was added sodium triacetoxyborohydride (4.77g, 22.5 mmol) and the reaction stirred overnight. The reaction mixture was washed (saturated sodium bicarbonate solution), dried ($MgSO_4$) and concentrated *in vacuo* to give a crude residue which was purified by column chromatography, eluting with 0-100%

EtOAc/DCM to give 5-bromo-N3-cyclohexylpyridine-2,3-diamine (2.54g, 63%) as a pale purple solid.

^{1}H NMR (500 MHz, DMSO--d_6): δ 7.25 (d, *J* = 2.0 Hz, 1H), 6.63 (d, *J* = 2.0 Hz, 1H), 5.72 (s, 2H), 4.77 (d, *J* = 7.5 Hz, 1H), 3.19 (m, 1H), 1.93 (m, 2H), 1.73 (m, 2H), 1.62 (m, 1H), 1.35 (m, 2H), 1.17 (m, 3H).

LRMS (ES+): *m/z* (%) 270.0 [^{79}Br M+H]$^+$,(100), 272.0 [^{81}Br M+H]$^+$ (98)

To a stirred solution of 5-bromo-N3-cyclohexylpyridine-2,3-diamine (168-012-001) (2.54g, 9.41 mmol) in MeCN (60 mL) was added CDI (6.1g, 37.6 mmol) and the reaction was stirred overnight. The solvent was removed *in vacuo* to give a crude residue which was triturated (water, diethyl ether) and dried *in vacuo* to give the title compound (**50a**) (2.08g, 75%) as a white solid.

^{1}H NMR (500 MHz, DMSO--d_6); δ 11.72 (brs, 1H), 7.99 (d, *J* = 1.9 Hz, 1H), 7.40 (d, *J* = 1.9 Hz, 1H), 4.14 (m, 1H), 2.06 (m, 2H), 1.82 (m, 2H), 1.70 (m, 2H), 1.64 (m, 1H), 1.17 (m, 3H).

LRMS (ES+): *m/z* (%) 296.0 [^{79}Br M+H]$^+$ (100) 298.0 [^{81}Br M+H]$^+$ (95)

6-bromo-1-(2-phenylpropan-2-yl)-1H-imidazo[4,5-b]pyridin-2(3H)-one 50b

To a stirred suspension of 2,3-amino-5-bromopyridine (1.13g, 6 mmol) in THF (20 mL) was added α,α–dimethylbenzyl chloride (1.02g, 6.6 mmol) and triethylamine (1.69 mL, 12 mmol) and the reaction was stirred for 66 hours. The solvent was removed *in vacuo* to give a crude residue which was purified by column chromatography, eluting with 0-40% EtOAc/hexane to give 5-bromo-N3-(2-phenylpropan-2-yl)pyridine-2,3-diamine (168 mg, 9%) as an off-white solid.

^{1}H NMR (500 MHz, $CDCl_3$): δ 7.55 (d, *J* = 2.0 Hz, 1H), 7.43 (m, 1H), 7.42 (m, 1H), 7.37 (m, 2H), 7.29 (m, 1H), 6.32 (d, *J* = 2.0 Hz, 1H), 4.17 (brs, 2H), 3.78 (brs, 1H), 1.70 (s, 6H)

LRMS (ES+): *m/z* (%) 306.0 [^{79}Br M+H]$^+$ (100), 308.0 [^{81}Br M+H]$^+$ (98)

To a stirred solution of 5-bromo-N3-(2-phenylpropan-2-yl)pyridine-2,3-diamine (168-004-001) (168 mg, 0.55 mmol) in MeCN (6 mL) was added CDI (267 mg, 1.65 mmol) and the reaction was stirred overnight. The solvent was removed *in vacuo* to give a crude residue which was triturated (water, diethyl ether) and dried *in vacuo* to give the title compound (**50b**) (127 mg, 69%) as a white solid.

^{1}H NMR (500 MHz, DMSO--d_6); δ 11.77 (brs, 1H), 7.89 (m); 7.40 (m, 4H), 7.35 (m, 1H), 6.32 (m, 1H), 1.98 (s, 6H)

LRMS (ES+): *m/z* (%) 332.0 [^{79}Br M+H]$^+$ (100), 334.0 [^{81}Br M+H]$^+$ (98).

1-cyclohexyl-6-phenyl-1H-imidazo[4,5-b]pyridin-2(3H)-one (51) (General method 6)

A capped process vial containing 6-bromo-1-cyclohexyl-1H-imidazo[4,5-b]pyridin-2(3H)-one (148 mg, 0.5 mmol), benzeneboronic acid pinacol ester (130 mg, 0.55mol), potassium phosphate (0.55 mL of a 1M solution in water, 0.55 mmol) and tetrakis(triphenylphoshine)palladium (0) (29 mg, 5mol%) in DMF (2 mL) was degassed, flooded with argon and heated under microwave irradiation at 130°C for 30 minutes. The reaction was diluted with DCM, washed (water, brine) and concentrated *in vacuo.* The resultant crude residue was purified by column chromatography, eluting with 0-20% methanol/DCM, followed by reverse-phase HPLC, to give the title compound (**51**) (45.3 mg, 31%) as a white solid.

^{1}H NMR (500 MHz, DMSO--d_6):δ 11.53 (brs, 1H), 8.18 (d, *J* = 1.9 Hz, 1H), 7.82 (d, *J* = 1.9 Hz, 1H), 7.71 (m, 2H), 7.49 (m, 2H), 7.38 (m, 1H), 4.23 (m, 1H), 2.16 (m, 2H), 1.85 (m, 2H), 1.73 (m, 2H), 1.67 (m, 1H), 1.38 (m, 3H).

LRMS (ES+): *m/z* (%) 294.2 [M+H]$^+$ (100)

HRMS (*m/z*): [MH+] calcd for C18H20N3O, 294.1601; found 294.1587

1-(2-phenylpropan-2-yl)-6-(pyridin-3-yl)-1H-imidazo[4,5-b]pyridin-2(3H)-one (52)

Following the method outlined in General method 6, but using 6-bromo-1-(2-phenylpropan-2-yl)-1H-imidazo[4,5-b]pyridin-2(3H)-one (63 mg, 0.191 mmol) and 3-pyridineboronic acid (26 mg, 0.21 mmol), the title compound (**52**) (5.3 mg, 3%) was obtained as a white solid.

^{1}H NMR (500 MHz, DMSO--d_6): δ 11.68 (brs, 1H), 8.50 (dd, *J* = 1.4 and 3.5 Hz, 1H), 8.47 (d, *J* = 2.1 Hz, 1H), 7.72 (m, 1H), 7.46 (m, 2H), 7.41 (m, 3H), 7.34 (m, 1H), 6.41 (d, *J* = 1.9 Hz); 2.06 (s, 6H)

LRMS (ES+): *m/z* (%) 331.1 [M+H]$^+$ (100)

HRMS (*m/z*): [MH+] calcd for C20H19N4O, 331.1553; found 331.1540

1-cyclohexyl-6-(2-(hydroxymethyl)phenyl)-1H-imidazo[4,5-b]pyridin-2(3H)-one (53)

Following the method outlined in General method 6, but using (2-hydroxymethyl)benzeneboronic acid (84 mg, 0.55 mmol), the title compound (**53**) (112 mg, 69%) was obtained as a yellow solid.

^{1}H NMR (500 MHz, DMSO--d_6): δ 11.61 (brs, 1H), 7.88 (d, *J* = 1.7 Hz, 1H), 7.73 (d, *J* = 1.7 Hz, 1H), 7.58 (d, *J* = 7.5 Hz, 1H), 7.41 (m, 1H), 7.36 (m, 1H), 7.1 (m, 1H), 5.19 (t, *J* = 5.2 Hz); 4.42 (d, *J* = 5.2 Hz); 4.21 (m, 1H), 2.09 (m, 2H), 1.83 (m, 2H), 1.74 (m, 2H), 1.63 (m, 1H), 1.35 (m, 3H).

LRMS (ES+): *m/z* (%) 324.2 $[M+H]^+$ (100)

HRMS (*m/z*): [MH+] calcd for C19H22N3O2, 324.1707; found 324.1693

6-(2-(hydroxymethyl)phenyl)-1-(2-phenylpropan-2-yl)-1H-imidazo[4,5-b]pyridin-2(3H)-one (54)

Following the method outlined in General method 6, but using 6-bromo-1-(2-phenylpropan-2-yl)-1H-imidazo[4,5-b]pyridin-2(3H)-one (63 mg, 0.191 mmol) and 2-(hydroxymethyl)phenylboronic acid (32 mg, 0.21 mmol), the title compound (**54**) (5.3 mg, 3%) was obtained as a white solid.

^{1}H NMR (500 MHz, DMSO--d_6): δ 11.59 (brs, 1H), 7.80 (d, J = 1.8 Hz); 7.48 (d, *J* = 7.6 Hz, 1H), 7.41-7.20 (m, 7H), 6.98 (dd, *J* = 1.1 and 7.6 Hz, 1H), 6.38 (d, *J* = 1.9 Hz); 5.02 (t, *J* = 5.5 Hz, 1H), 4.11 (d, J = 5.3 Hz, 2H), 2.00 (s, 6H)

LRMS (ES+): *m/z* (%) 360.2 $[M+H]^+$ (100)

1-cyclohexyl-6-(pyridin-3-yl)-1H-imidazo[4,5-b]pyridin-2(3H)-one (55)

Following the method outlined in General method 6, but using 3-pyridineboronic acid (37 mg, 0.55 mmol), the title compound (**55**) (95 mg, 68%) was obtained as a yellow solid.

^{1}H NMR (500 MHz, DMSO--d_6): δ 11.66 (brs, 1H), 8.95 (dd, *J* = 0.7 and 1.7 Hz, 1H), 8.59 (dd, *J* = 1.5 and 3.3 Hz, 1H), 8.25 (d, *J* = 1.9 Hz, 1H), 8.14 (m, 1H), 7.93 (d, *J* = 1.9 Hz, 1H), 7.50 (m, 1H), 4.23 (m, 1H), 2.19 (m, 2H), 1.86 (m, 2H), 1.74 (m, 2H), 1.66 (m, 1H), 1.39 (m, 3H).

LRMS (ES+): *m/z* (%) 295.1 $[M+H]^+$ (100)

HRMS (*m/z*): [MH+] calcd for C17H19N4O, 295.1553; found 295.1541

1-cyclohexyl-6-(piperidin-1-yl)-1H-imidazo[4,5-b]pyridin-2(3H)-one (56) (General method 7)

A capped process vial containing 6-bromo-1-cyclohexyl-1H-imidazo[4,5-b]pyridin-2(3H)-one (148 mg, 0.5 mmol), piperidine (99μL, 1 mmol), tris(dibenzylideneacetone)dipalladium(0) (46 mg, 10mol%), (*S*)-PHOS (41 mg, 0.1 mmol) and sodium t-butoxide (144 mg, 1.5 mmol) in toluene (4 mL) was heated under microwave irradiation at 120°C for 20 minutes. The reaction was diluted with DCM, washed (saturated sodium bicarbonate) and concentrated *in vacuo.* The resultant crude residue was purified by column chromatography on KP-NH silica, eluting with 0-10% MeOH/EtOAc, followed by reverse-phase HPLC, to give the title compound (**56**) (65 mg, 43%) as a white solid.

^{1}H NMR (500 MHz, DMSO--d_6): δ 11.13 (brs, 1H), 7.54 (d, *J* = 2.2 Hz, 1H), 7.25 (d, *J* = 2.2 Hz); 4.13 (m, 1H), 3.05 (m, 4H), 2.08 (m, 2H), 1.83 (m, 2H), 1.66 (m, 6H), 1.52 (m, 2H), 1.39 (m, 4H).

LRMS (ES+): *m/z* (%) 301.2 $[M+H]^+$ (100)

HRMS (*m/z*): [MH+] calcd for C17H25N4O, 301.2023; found 301.2008

1-cyclohexyl-6-morpholino-1H-imidazo[4,5-b]pyridin-2(3H)-one (57)

Following the method outlined in General method 7, but using morpholine (87μL, 1 mmol), the title compound (**57**) (56 mg, 37%) was obtained as a white solid.

^{1}H NMR (500 MHz, DMSO--d_6): δ 11.18 (brs, 1H), 7.55 (d, *J* = 2.3 Hz, 1H), 7.28 (d, *J* = 2.3 Hz); 4.14 (m, 1H), 3.76 (m, 4H), 3.08 (m, 4H), 2.09 (m, 2H), 1.84 (m, 2H), 1.70 (m, 3H), 1.41 (m, 3H).

LRMS (ES+): *m/z* (%) 303.2 $[M+H]^+$ (100)

HRMS (*m/z*): [MH+] calcd for C16H23N4O2, 303.1816; found 303.1803

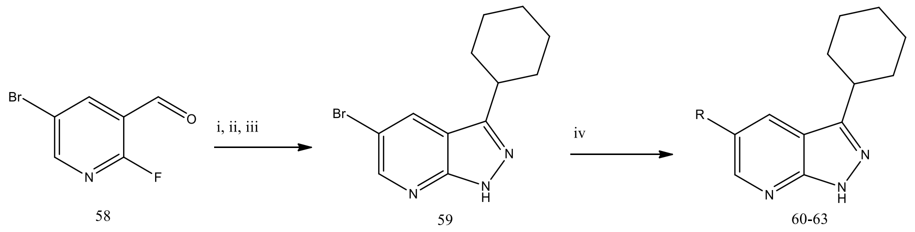

Scheme 7: i) Cyclohexylmagnesium bromide, THF, -78°C to rt, 55%; ii) PDC, DCM, 0°C to rt, 96%; iii) $NH_2NH_2.H_2O$, EtOH, reflux, 95%; iv) $RB(OH)_2$, 2M Na_2CO_3, $Pd(PPh_3)_4$, DME:H_2O:EtOH (7:3:2), 140°C, microwave, 16-69%.

5-bromo-3-cyclohexyl-1H-pyrazolo[3,4-b]pyridine (59)

To a stirred solution of 5-bromo-2-fluoro-3-formylpyridine (3.06g, 15 mmol) in THF (65 mL) at -78°C, under an inert atmosphere was added 1M cyclohexylmagnesium bromide in THF (22.5 mL, 22.5 mmol) dropwise, the reaction warmed to room temperature and stirred for 16 hours. The reaction was quenched (saturated ammonium chloride solution), the layers separated and the aqueous layer extracted (EtOAc). The combined organics were washed (brine), dried ($MgSO_4$) and concentrated *in vacuo*. The crude residue was purified by column chromatography, eluting with 0-20% EtOAc/hexane to give (5-bromo-2-fluoropyridin-3-yl)(cyclohexyl)methanol (168-016-001) (1.68g, 55%) as a yellow oil.

^{1}H NMR (500 MHz, DMSO--d_6): δ 8.24 (brd, J = 0.9 Hz, 1H), 8.09 (dd, J = 2.2 and 5.8 Hz); 5.51 (d, J = 4.90 Hz, 1H), 4.48 (m, 1H), 1.70 (m, 3H), 1.56 (m, 2H), 1.38 (m, 1H), 1.09 (m, 5H).

LRMS (ES+): *m/z* (%) 288.0 [^{79}Br M+H]$^+$, (100), 290.0 [^{81}Br M+H]$^+$ (98)

To a stirred suspension of Pyridinium dichromate (5.49g, 14.6 mmol) in DCM (15 mL) at 0°C, under an inert atmosphere, was added (5-bromo-2-fluoropyridin-3-yl)(cyclohexyl)methanol (1.68g, 5.83 mmol) in DCM (5 mL), the reaction warmed to room temperature and stirred for 66 hours. Additional Pyridinium dichromate (5.49g, 14.6 mmol) was added and stirring continued for 24 hours. The reaction mixture was filtered through a pad of HM-N sorbent and the filtrate was concentrated *in vacuo* to give a crude residue which was purified by column chromatography, eluting with 0-10% EtOAc/hexane, to give (5-bromo-2-fluoropyridin-3-yl)(cyclohexyl)methanone (1.52g, 96%) as a straw oil.

^{1}H NMR (500 MHz, DMSO--d_6): δ 8.60 (m, 1H), 8.51 (dd, *J* = 2.5 and 5.8 Hz, 1H), 3.17 (m, 1H), 1.84 (m, 2H), 1.74 (m, 2H), 1.65 (m, 1H), 1.40-1.09 (m, 5H).

LRMS (ES+): *m/z* (%) 286.0 [^{79}Br M+H]$^+$(100), 288.0 [^{81}Br M+H]$^+$ (98)

To a stirred solution of (5-bromo-2-fluoropyridin-3-yl)(cyclohexyl)methanone (1.51g, 5.28 mmol) in EtOH (20 mL) was added hydrazine hydrate (770μL, 15.8 mmol), the reaction heated to reflux and stirred for 16 hours. The solvent was removed *in vacuo* to give the title compound (**59**) (1.41g, 95%) as a white solid.

^{1}H NMR (500 MHz, DMSO--d_6): δ 13.36 (brs, 1H), 8.60 (m,1H), 8.54 (m, 1H), 3.04 (m, 1H), 1.96 (m, 2H), 1.82 (m, 2H), 1.67 (m, 3H), 1.38 (m, 3H).

LRMS (ES+): *m/z* (%) 280.0 [^{79}Br M+H]$^+$(100), 282.0 [^{81}Br M+H]$^+$ (98)

3-cyclohexyl-5-phenyl-1H-pyrazolo[3,4-b]pyridine (60) (General method 8)

A capped process vial containing 5-bromo-3-cyclohexyl-1H-pyrazolo[3,4-b]pyridine (140 mg, 0.5 mmol), benzeneboronic acid pinacol ester (142 mg, 0.6mol), sodium carbonate (0.38 mL of a 2M solution in water, 0.76 mmol) and tetrakis(triphenylphoshine)palladium (0) (12 mg, 2mol%) in 7:3:2 1,2-dimethoxyethane:water:ethanol (3 mL) was degassed, flooded with argon and heated under microwave irradiation at 140°C for 35 minutes. The reaction was diluted with DCM, washed (saturated sodium bicarbonate) and concentrated *in vacuo.* The resultant crude residue was purified by column chromatography on KP-NH silica, eluting with 0-100% EtOAc/DCM, followed by reverse-phase HPLC, to give the title compound (**60**) (88 mg, 63%) as a white solid.

^{1}H NMR (500 MHz, DMSO--d_6): δ 13.23 (brs, 1H), 8.78 (d, *J* = 1.9 Hz, 1H), 8.50 (d, *J* = 2.0 Hz, 1H), 7.80 (m, 2H), 7.51 (m, 2H), 7.41 (m, 1H), 3.11 (m, 1H), 2.04 (m, 2H), 1.84 (m, 2H), 1.72 (m, 3H), 1.40 (m, 3H).

LRMS (ES+): *m/z* (%) 278.2 [M+H]$^+$ (100)

HRMS (*m/z*): [MH+] calcd for C18H20N3, 278.1652; found 278.1640

(2-(3-cyclohexyl-1H-pyrazolo[3,4-b]pyridin-5-yl)phenyl)methanol (61)

Following the method outlined in General method 8, but using (2-hydroxymethyl)benzeneboronic acid (91 mg, 0.6 mmol), the title compound (**61**) (25 mg, 16%) was obtained as a white solid.

^{1}H NMR (500 MHz, DMSO--d_6): δ 13.27 (brs, 1H), 8.48 (d, *J* = 2.1 Hz, 1H), 8.32 (d, *J* = 2.0 Hz, 1H), 7.60 (d, *J* = 7.7 Hz, 1H), 7.38 (m, 3H), 5.21 (t, *J* = 5.3 Hz, 1H), 4.41 (d, *J* = 5.2 Hz, 2H), 3.05 (m, 1H), 2.00 (m, 2H), 1.81 (m, 2H), 1.68 (m, 3H), 1.38 (m, 3H).

LRMS (ES+): *m/z* (%) 308.2 $[M+H]^+$ (100)

HRMS (*m/z*): [MH+] calcd for C19H22N3O, 308.1757; found 308.1741

3-cyclohexyl-5-(2-(methoxymethyl)phenyl)-1H-pyrazolo[3,4-b]pyridine (62)

Following the method outlined in General method 8, but using (2-methoxymethyl)benzeneboronic acid (100 mg, 0.6 mmol), the title compound (**62**) (111 mg, 69%) was obtained as a white solid.

^{1}H NMR (500 MHz, DMSO--d_6): δ 13.26 (brs, 1H), 8.48 (d, *J* = 1.9 Hz, 1H), 8.31 (d, *J* = 1.9 Hz, 1H), 7.55 (m, 1H), 7.44 (m, 2H), 7.40 (m, 1H), 4.29 (s, 2H), 3.30 (s, 3H), 3.05 (m, 1H), 2.01 (m, 2H), 1.82 (m, 2H), 1.69 (m, 3H), 1.38 (m, 3H).

LRMS (ES+): *m/z* (%) 322.2 $[M+H]^+$ (100)

HRMS (*m/z*): [MH+] calcd for C20H24N3O, 322.1914; found 322.1901

3-cyclohexyl-5-(pyridin-3-yl)-1H-pyrazolo[3,4-b]pyridine (63)

Following the method outlined in General method 8, but using 3-pyridineboronic acid (74 mg, 0.6 mmol), the title compound (**63**) (74 mg, 53%) was obtained as a white solid.

^{1}H NMR (500 MHz, DMSO--d_6): δ 13.08 (brs, 1H), 8.81 (m, 1H), 8.61 (d, *J* = 2.0 Hz, 1H), 8.41 (d, *J* = 2.0 Hz, 1H), 8.38 (dd, *J* = 1.5 and 3.2 Hz, 1H), 8.00 (m, 1H), 7.33 (m, 1H), 2.90 (m, 1H), 1.82 (m, 2H), 1.64 (m, 2H), 1.49 (m, 3H), 1.20 (m, 3H).

LRMS (ES+): *m/z* (%) 279.2 $[M+H]^+$ (100)

HRMS (*m/z*): [MH+] calcd for C17H19N4, 279.1604; found 279.1591

S2. Kinase Profiling data

(blue)	% of activity higher than 30% respect of the control
(red)	higher 90% inhibition
(peach)	between 80% and 90% of inhibition
(yellow)	between 80% and 70% of inhibition

ID	1	12	10	14
Structure	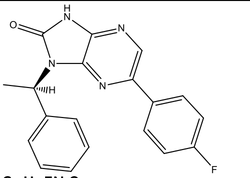 $C_{19}H_{15}FN_4O$	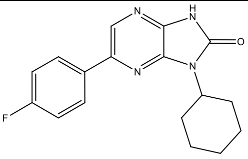 $C_{17}H_{17}FN_4O$	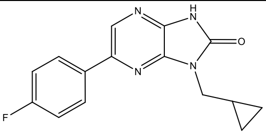 $C_{15}H_{13}FN_4O$	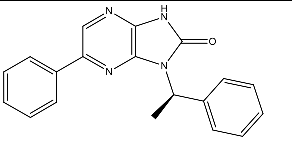 $C_{19}H_{16}N_4O$
MKK1	106	62	97	87
ERK1	153	115	112	105
ERK2	65	41	84	121
JNK1	95	95	100	97
JNK2	104	93	97	96
p38a MAPK	88	86	87	88
P38b MAPK	84	122	101	95
p38g MAPK	98	98	94	91
p38s MAPK	108	76	91	77
ERK8	73	80	70	79
RSK1	96	89	85	89
RSK2	110	104	113	101
PDK1	99	98	96	91
PKBa	99	102	96	89
PKBb	117	106	120	100
SGK1	104	111	110	98
S6K1	81	97	92	65
PKA	87	95	93	72
ROCK 2	105	84	78	85
PRK2	111	112	99	97
PKCa	101	94	106	98
PKC zeta	94	90	90	88
PKD1	55	60	40	44
MSK1	89	97	89	93
MNK1	83	93	93	76
MNK2	106	103	99	97
MAPKAP-K2	90	101	104	84
PRAK	103	99	99	98
CAMKKb	101	104	98	86
CAMK1	87	88	83	72
SmMLCK	80	89	74	64
PHK	103	110	97	94
CHK1	100	96	90	94
CHK2	97	96	97	93
GSK3b	63	70	37	66
CDK2-Cyclin A	89	84	82	84
PLK1	111	117	102	104
PLK1 (Okadaic Acid)	96	96	109	104
Aurora B	117	85	93	99
AMPK	97	100	94	95
MARK3	110	98	97	100
BRSK2	82	100	90	72
MELK	118	110	125	100
CK1	75	98	75	71
CK2	107	111	105	105
DYRK1A	79	93	113	85
DYRK2	100	99	94	89
DYRK3	109	99	108	100
NEK2a	117	93	101	96
NEK6	89	94	86	80
IKKb	103	94	105	77
PIM1	80	82	79	72
PIM2	113	111	111	96
PIM3	83	90	89	83
SRPK1	80	88	89	75
MST2	86	94	92	78
EF2K	93	98	92	79
HIPK2	101	97	69	80
PAK4	101	103	106	86
PAK5	84	100	95	80
PAK6	89	106	98	88
Src	80	94	98	70
Lck	90	105	99	89
CSK	100	90	93	77
FGF-R1	95	96	88	63
IRR	96	83	104	112
EPH A2	105	97	96	89
MST4	90	100	92	88
SYK	97	100	98	84
YES1	94	96	96	71
IKKe	106	88	94	91
TBK1	95	92	95	100
IGF-1R	122	100	85	112
VEG-FR	117	107	98	73
BTK	113	111	104	97
IR-HIS	88	119	109	93
EPH-B3	113	110	92	102
TBK1 (DU12569)	95	90	86	78
IKK epsilon (14231)	124	98	103	87

S3. STPH screen assay protocols

Activity against Trypanosoma brucei rhodesiense STIB900. This stock was isolated in 1982 from a human patient in Tanzania and after several mouse passages cloned and adapted to axenic culture conditions (Baltz et al 1985). Minimum Essential Medium (50 µl) supplemented with 25 mM HEPES, 1g/l additional glucose, 1% MEM non-essential amino acids (100x), 0.2 mM 2-mercaptoethanol, 1mM Na-pyruvate and 15% heat inactivated horse serum was added to each well of a 96-well microtiter plate. Compounds were dissolved in 30% DMSO at 3 mM and further diluted with MEM to 100 μM. 5 μl and 1 μl of the compound solution respectively were added to the wells, resulting in a test concentration of 5μM and 1μM. One assay plate was prepared per test concentration. Then $4x10^3$ bloodstream forms of *T. b. rhodesiense* STIB 900 in 50 µl was added to each well and the plate incubated at 37 °C under a 5 % CO_2 atmosphere for 70 h. 10 µl resazurin solution (resazurin, 12.5 mg in 100 ml double-distilled water) was then added to each well and incubation continued for a further 2–4 h (Raz et al 1997). Then the plates were read with a Spectramax Gemini XS microplate fluorometer (Molecular Devices Cooperation, Sunnyvale, CA, USA) using an excitation wave length of 536 nm and an emission wave length of 588 nm. The data were evaluated in Excel. For each test concentration, the percent growth inhibition was calculated in comparison with an untreated control.

Baltz, T., D. Baltz, C. Giroud, and J. Crockett. 1985. Cultivation in a semi-defined medium of animal infective forms of Trypanosoma brucei, T. equiperdum, T. evansi, T. rhodesiense and T. gambiense. EMBO Journal 4:1273-1277.

Räz, B., M. Iten, Y. Grether-Buhler, R. Kaminsky, and R. Brun. 1997. The Alamar Blue assay to determine drug sensitivity of African trypanosomes (T.b. rhodesiense and T.b. gambiense) in vitro. Acta Trop 68:139-47.

In vitro cytotoxicity with L-6 cells. Assays were performed in 96-well microtiter plates, each well containing 100 μl of RPMI 1640 medium supplemented with 1% L-glutamine (200mM) and 10% fetal bovine serum, and 4000 L-6 cells (a primary cell line derived from rat skeletal myoblasts)(Page et al., 1993 and Ahmed et al., 1994). Compounds were dissolved in 30% DMSO at 3 mM and further diluted with MEM to 100 μM. 5 μl and 1 μl of the compound solution respectively were added to the wells, resulting in a test concentration of 5μM and 1μM. One assay plate was prepared per test concentration. After 70hours of incubation at 37

°C under a 5 % CO_2 atmosphere the plates were inspected under an inverted microscope to assure growth of the controls and sterile conditions. 10 µl resazurin solution (resazurin, 12.5 mg in 100 ml double-distilled water) was then added to each well and incubation continued for a further 2h. Then the plates were read with a Spectramax Gemini XS microplate fluorometer (Molecular Devices Cooperation, Sunnyvale, CA, USA) using an excitation wave length of 536 nm and an emission wave length of 588 nm. The data were evaluated in Excel. For each test concentration, the percent growth inhibition was calculated in comparison with an untreated control.

C.Page C, M. Page and C. Noel. 1993. A new fluorimetric assay for cytotoxicity measurements in vitro.International Journal of Oncology 3: 473–476.

S.A. Ahmed, R.M. Gogal and J.E. Walsh. 1994. A new rapid and simple non-radioactive assay to monitor and determine the proliferation of lymphocytes: an alternative to [3H] thymidine incorporation assay. The Journal of Immunological Methods 170: 211–224.

Definition of test score:

If growth inhibition at higher concentration is <50% the compound is classified as not toxic

Hit rate details from MTS screen of kinase set

Table 1 Numbers of hits and hit rate depending on activity criteria

activity criteria	*T.b. rhod.* (5µM)		*T.b. rhod.* (1µM)		Cytotoxicity (30µM)	
% growth inhibition	# hits	hit rate in %	# hits	hit rate in %	# hits	hit rate in %
≥ 50	121	3.1	29	0.7	131	3.4
≥ 60	95	2.4	21	0.5	78	2
≥ 70	78	2	19	0.5	47	1.2
≥ 80	63	1.6	16	0.4	34	0.9
Tested cpds	3885					

S4. Dose response and kill curves from *T.b.brucei.* static/cidal assay

Static-cidal EC_{50} at 48h

Static-cidal time course

24

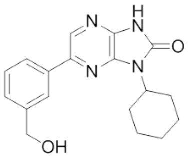

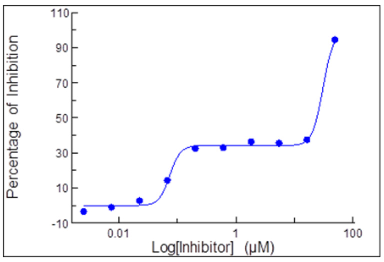

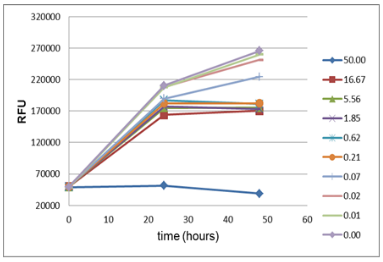

26

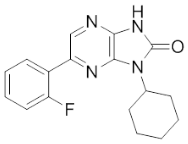

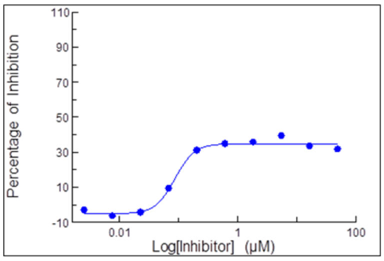

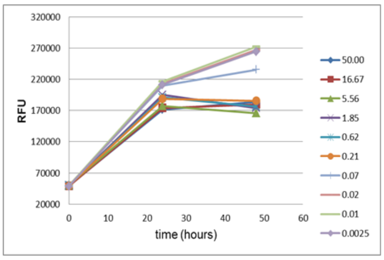

28

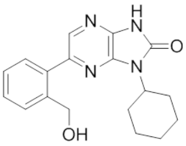

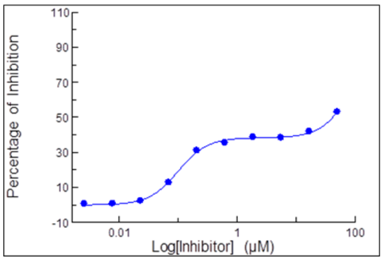

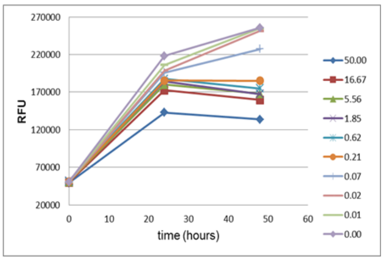

	Static-cidal EC_{50} at 48h	Static-cidal time course

30

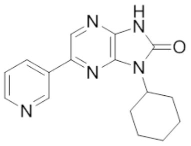

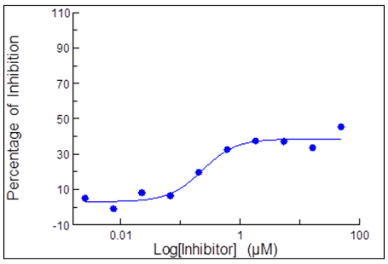

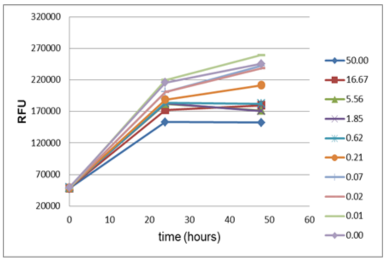

39

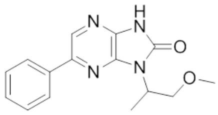

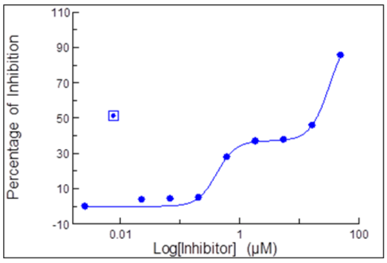

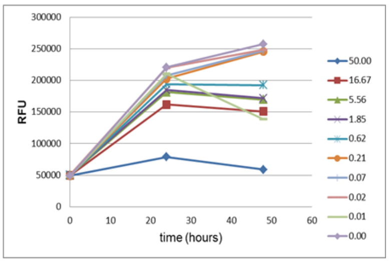

51

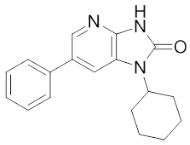

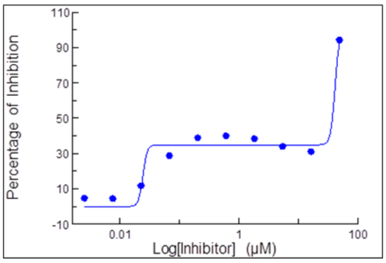

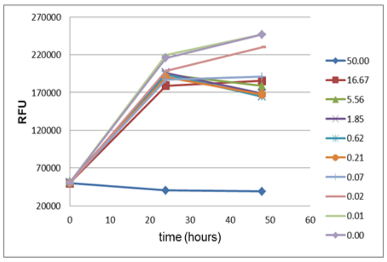

Static-cidal EC_{50} at 48h

Static-cidal time course

55

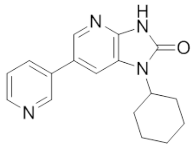

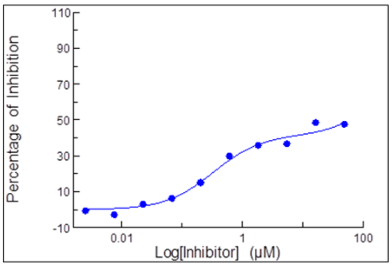

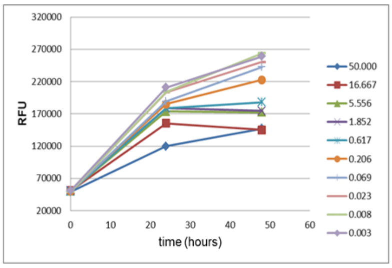

56

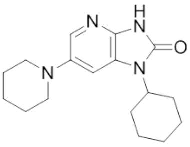

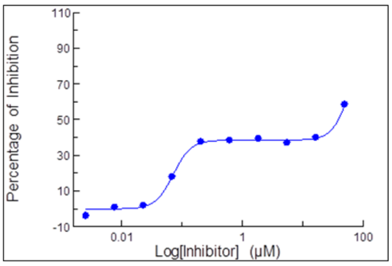

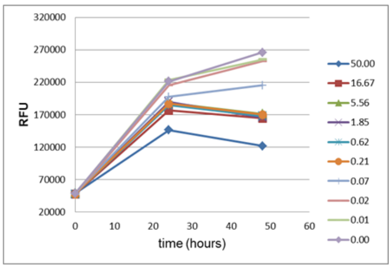

60

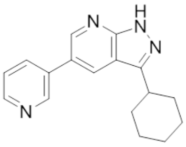

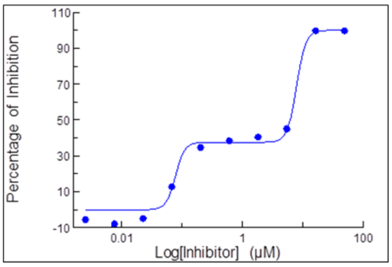

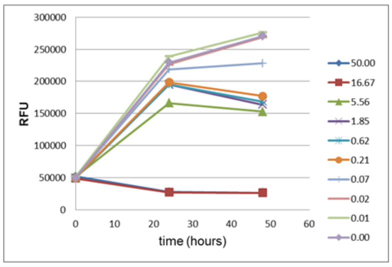

Static-cidal EC_{50} at 48h

Static-cidal time course

63

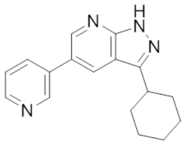

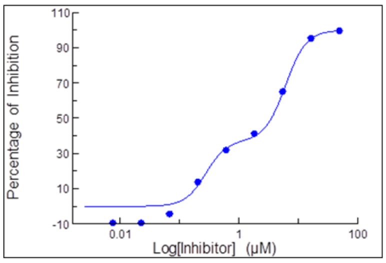

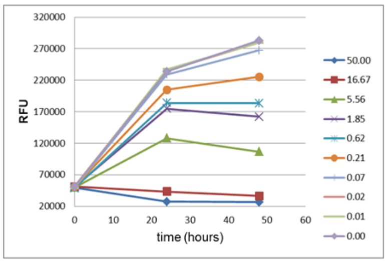

Pentamidine

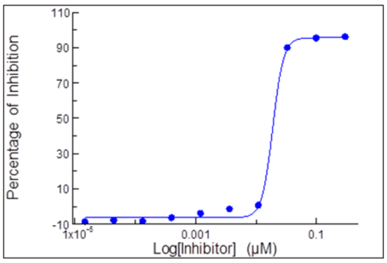

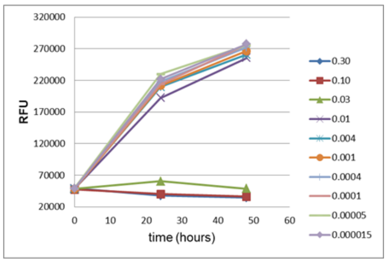

Melarsoprol

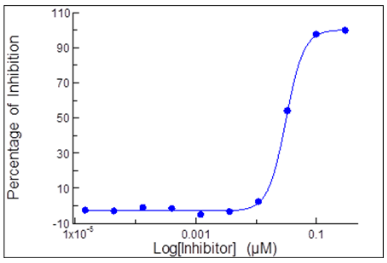

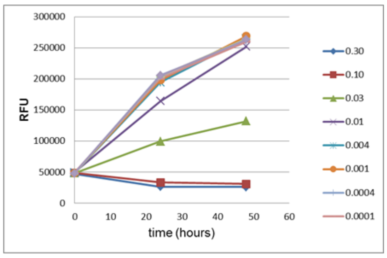

Supplement: Supplementary file 1 — Supplementary [file cmdc0010-1809-sd1.pdf]
